# Mapping and identifying service models for community-based services for children with intellectual disabilities and behaviours that challenge in England

**DOI:** 10.1186/s12913-023-10388-9

**Published:** 2023-12-04

**Authors:** Emma L. Taylor, Paul A. Thompson, Nicholas Manktelow, Samantha Flynn, David Gillespie, Jill Bradshaw, Nick Gore, Ashley Liew, Mark Lovell, Kate Sutton, Caroline Richards, Stavros Petrou, Peter E. Langdon, Gemma Grant, Vivien Cooper, Kate Seers, Richard P. Hastings

**Affiliations:** 1 Centre for Research in Intellectual and Developmental Disabilities (CIDD), University of Warwick, Coventry, CV4 8UW UK; 2 Centre for Trials Research, Cardiff University, Cardiff, CF14 4YS UK; 3 Tizard Centre, University of Kent, Canterbury, CT2 7NF UK; 4 Evelina London Children’s Hospital, Westminster Bridge Rd, London, SE1 7EH UK; 5 South London and Maudsley NHS Foundation Trust, Michael Rutter Centre, London, SE5 8AZ UK; 6 Tees, Esk and Wear Valleys, NHS Foundation Trust, Rosewood Centre, Acklam Road Hospital, Acklam Road, Middlesbrough, TS5 4EE UK; 7 Learning Disability and Autism Programme, NHS England, London, UK; 8 School of Psychology/ UK & Cerebra Network, University of Birmingham, Edgbaston, Birmingham, B15 2TT UK; 9 Nuffield Department of Primary Care Health Sciences, University of Oxford, Oxford, UK; 10 Coventry and Warwickshire Partnership NHS Trust, Coventry, UK; 11 Herefordshire and Worcestershire Health and Care NHS Trust, Worcester, UK; 12 The Challenging Behaviour Foundation, Chatham, Kent, ME4 6BE UK; 13 Warwick Medical School, University of Warwick, Coventry, CV4 7AL UK

**Keywords:** Children, Intellectual disabilities, Learning disabilities, Behaviours that challenge, Challenging behaviour, Community services, Service models, Mapping, NHS

## Abstract

**Background:**

One in five children with an intellectual disability in the UK display behaviours that challenge. Despite associated impacts on the children themselves, their families, and services, little research has been published about how best to design, organise, and deliver health and care services to these children. The purpose of this study was to describe how services are structured and organised (“service models”) in England for community-based health and care services for children with intellectual disability who display behaviours that challenge.

**Methods:**

Survey data about services were collected from 161 eligible community-based services in England. Staff from 60 of these services were also interviewed. A combination of latent class and descriptive analysis, coupled with consultation with family carers and professionals was used to identify and describe groupings of similar services (i.e., “service models”).

**Results:**

The latent class analysis, completed as a first step in the process, supported a distinction between specialist services and non-specialist services for children who display behaviours that challenge. Planned descriptive analyses incorporating additional study variables were undertaken to further refine the service models.

Five service models were identified: Child and Adolescent Mental Health Services (CAMHS) (*n =* 69 services), Intellectual Disability CAMHS (*n =* 28 services), Children and Young People Disability services (*n =* 25 services), Specialist services for children who display behaviours that challenge (*n =* 27 services), and broader age range services for children and/or adolescents and adults (*n=* 12 services).

**Conclusions:**

Our analysis led to a typology of five service models for community health and care services for children with intellectual disabilities and behaviours that challenge in England. Identification of a typology of service models is a first step in building evidence about the best provision of services for children with intellectual disabilities who display behaviours that challenge. The methods used in the current study may be useful in research developing service typologies in other specialist fields of health and care.

**Study registration:**

Trial Registration: Current Controlled Trials ISRCTN88920546, Date assigned 05/07/2022.

**Supplementary Information:**

The online version contains supplementary material available at 10.1186/s12913-023-10388-9.

## Background

Intellectual Disability is characterised by impairments in cognitive functioning with an IQ of 69 or below, and reduced levels of adaptive behaviour such as communication, social and independence skills, with an onset during childhood or adolescence (the developmental period) [1]. Around 2% of children in England have an intellectual disability [2].

Approximately 1 in 5 children with intellectual disabilities in the UK who are in contact with services display behaviours that challenge [3] although prevalence rates are higher in some settings such as special schools [4]. Behaviours that challenge are socially defined in terms of their impact. They are culturally atypical behaviours and occur at a frequency, severity, or duration that leads to a negative impact upon the person themselves (e.g., self-injury, exclusion, poorer care outcomes, personal safety) or those around them including families (e.g., stress, physical harm) [5, 6]. Behaviours that challenge can also lead to increased care costs for families [7], and health and social care services [8].

Given the prevalence of behaviours that challenge and continued high profile care scandals (e.g., at Winterbourne View and Whorlton Hall [9, 10]), effective community-based services and supports are a national priority [11]. However, there was no high-quality evidence relating to the design and organisation of services for children who have behaviours that challenge identified within National Institute for Health and Care Excellence (NICE) guidelines [12, 13]. In fact, a specific recommendation within NICE NG93 guideline [13] was for researchers to explore what models of support are effective and cost-effective for people with intellectual disabilities who display behaviours that challenge.

There are currently no England-wide data about how health and care services are organised for children with intellectual disabilities who display behaviours that challenge (service models), the key features of these models, and the outcomes and costs associated with different models or characteristics of services. Although co-production of the design and delivery of services with families (potentially including children) is recommended (e.g., NICE, [13]), there are also no data on how services may be delivering on these recommendations, nor the outcomes associated with different approaches to co-production (including family carer satisfaction, and costs). There is recent data about services for adults with intellectual disabilities and behaviours that challenge. Hassiotis et al. [14, 15] mapped 73 Intensive Support Teams in the UK and determined the typology of service models, using both cluster analysis and thematic analysis, while excluding broader community-based services. They identified two models: (1) independent (stand-alone teams), and (2) enhanced provision based around a community intellectual disability service. No similar exercise has been undertaken for community services for children with intellectual disability and behaviours that challenge and high-quality similar data help inform future commissioning and delivery of services to support children with intellectual disability and behaviours that challenge and their families. Therefore, the aim of this study was to conduct a mapping exercise to describe English community-based services for children and young people with intellectual disability who display behaviours that challenge, and to develop a typology of “service models”.

## Methods

### Research design

Eligible services were geographically located in England and at least partially drew referrals from England. All eligible services either: provided support for children and young people aged between birth and 17 years with intellectual disability who display behaviours that challenge; or provided supports to this group of children and young people as a clearly distinct care pathway (whilst also providing other services). Specific services types considered were: (1) community-based National Health Service (NHS), local authority (education or social care) or other (e.g., private, charity) services; (2) commissioned by a Clinical Commissioning Group (CCG)/local authority (education or social care)/Sustainability and Transformation Partnership/Integrated Care System/Boards (ICS/B), or (3) a service where individual places are purchased by a CCG/local authority or other commissioner.

Provision of support to people over 17 years of age was not a reason for exclusion from this study, provided services also supported children and young people who were 17 years of age or under. Services were excluded if they were: (1) an inpatient service; (2) commissioned by a non-CCG or local authority commissioner (e.g., solely a special school service); or (3) not yet operational (i.e., had received no referrals at the time of data collection).

The initial plan was to gather data solely through interviews with services. Key question areas for interviews were informed by consultation with clinicians and with the Challenging Behaviour Foundation, our Patient and Public Involvement partner. Later reflection on what would make the process most convenient for services led us to adapt data collection into a two-step process – with an initial survey containing key information about services that could be broken down into closed questions (with some limited use of free-text responses), and then a follow-up interview exploring information that could not be adequately captured in a survey (Additional file 1 for interview schedule, section "Conclusions").

The survey included categorical response options and free-text response questions about key dimensions including commissioning, staffing, caseloads, support provided, outcome measures used, referrals, individual characteristics of the child, and assessment/intervention approaches used (see additional file 1 for full survey questions, section 5). Information gathered in the interviews included the history of the service and future plans, connections to other local services, additional access and eligibility requirements, opportunities for children and families to be involved in decision-making and co-production, diversity and inclusion, and management and supervision structures.

Initially, there were 33 items in the survey, which was reduced to 23 items after interim data checks suggested that some survey items would not complement outputs from the Latent Class Analysis (LCA) and the research team was attempting to reduce survey completion time to encourage participation. Statistical problems were due mainly to low participant response rates, and variation in participant responses to some questions (e.g., if most participants responded in the same response category, there would be minimal variation).

### Participating services and staff

**Fig. 1:**
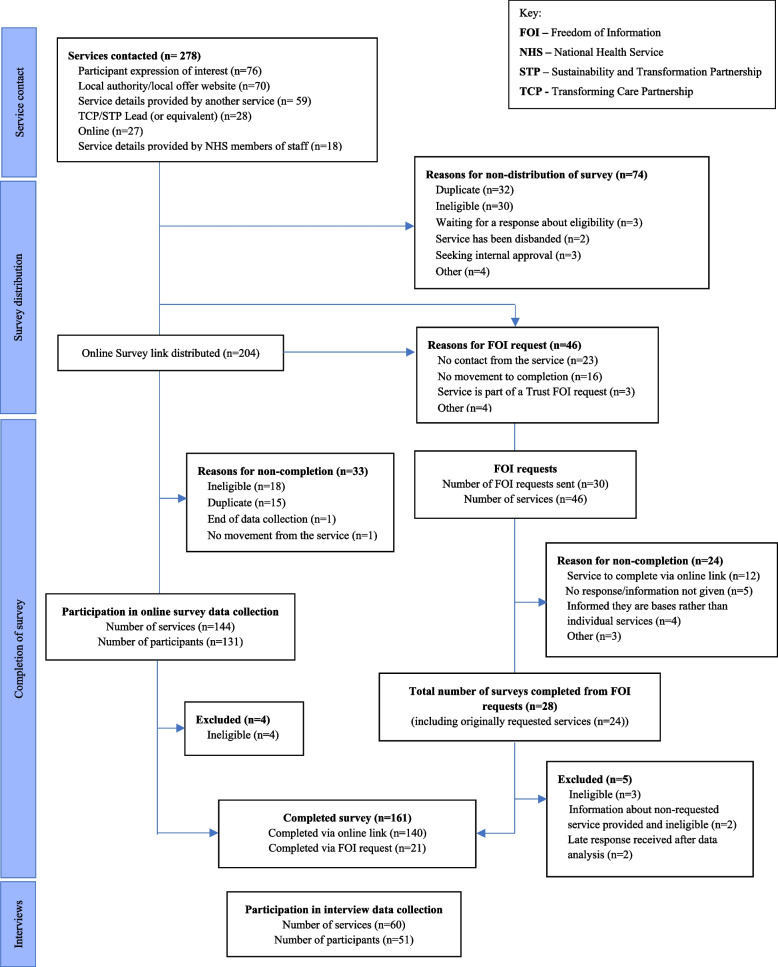
Strobe Flowchart of recruitment and data collection

A total of 278 services were contacted to take part in the study, with 204 survey links distributed to services thought to be eligible. Survey data were collected from 161 eligible services, with 60 of those services also completing an interview. Figure 1 summarises the recruitment and data collection process. A total of 125 participants took part in the survey to provide information about their service. 47 respondents held managerial responsibility (e.g., service managers *n=*12, team managers/leads *n=*19, clinical leads *n=*16), whilst 56 respondents worked in clinical roles (e.g., clinical psychologist *n=*24, nurse practitioner *n=*11, psychologist *n=*3, psychiatrist *n=*10, matron *n=*2). Some 22 respondents held joint managerial and clinical responsibilities.

### Procedure

Potentially eligible services were identified through a number of methods, including contacting all 48 Transforming Care Partnerships (TCPs) (or successor structures at the time) in England about community services for children with intellectual disability and behaviours that challenge in their region, direct contact with NHS Trust Research and Development departments, regional and national NHS England contacts, newsletters/mailing lists, word-of-mouth, online (e.g., social media, email distribution lists) distribution of study information, and collecting published information from local authority “Local Offer” special educational needs websites. All potentially eligible services were contacted by the study team with information about the study, and an invitation to participate followed if the service expressed initial interest. The research team identified a service as a duplicate if more than one member of staff from the same service had expressed interest in taking part in the study. Ethical approval was obtained from the Humanities and Social Sciences Research Ethics Committee at the University of Warwick (REF: HSSREC 91/20-21), prior to any data collection.

Services were assessed for eligibility against an eligibility checklist during a telephone call or via email. Services identified as ineligible by the research team were excluded from taking part in the study. Eligible services were asked to identify staff members to take part in the online survey and/or interview with a researcher. The research team suggested that service managers and clinical leads would be well placed to answer questions, but services decided the most appropriate person to complete the survey.

All stages of the study procedures were piloted with three eligible services before data collection began. This process confirmed that the study procedures were feasible and acceptable to services. For the survey, piloting highlighted some presentation/online survey set-up issues that were then corrected and led to the inclusion of additional multiple-choice response options to questions concerning type of staff, as the services were not able to categorise some of their staff using the existing response options. For the interviews, piloting resulted in an additional question about sub-teams within services being added in response to the service making a distinction between their sub-teams. Pilot data were included in the main statistical analysis as the items included remained unchanged, the services were eligible, and their inclusion in the analysis maximised the sample of services analysed.

After a service expressed initial interest, they were sent an email containing an information sheet, a PDF copy of the survey for information, and a personalised survey link that included a copy of the information sheet and a consent form. Staff from services could complete the survey online themselves or with a researcher via Microsoft Teams® or telephone, or by entering the data onto the PDF document and returning to the research team by post or email. Consent was sought prior to completion of the survey or interview, and additionally confirmed verbally at the start of interviews. Online survey and interview responses were managed using the Qualtrics**©** survey platform and data were downloaded weekly onto a secure server and pseudonymised before data analysis began.

Services that expressed interest but did not respond to communications after they confirmed they were seeking approval internally to take part in the study were not sent a personalised survey link. On average, the research team made four contact attempts with these services via email and telephone. Following an ethics amendment in October 2021, these services were sent a Freedom of Information (FOI) request to mitigate the risk of their data otherwise being missed from the study.

### Analysis

#### Main statistical analysis

Latent class analysis (LCA) is a data driven approach focusing on an individual’s responses, or in this study, the services, rather than a variable-centred approach to determine if homogeneous subgroups or classes are present [16]. The overarching benefit of the statistical approach is its ability to distinguish the groups using only the data without the influence of researcher’s assumptions or biases to inform the groupings. Patterns of scores from services were used to identify potential similarities, and as a means of grouping services using a probabilistic model. Multiple latent class models were fitted with increasing numbers of classes, up to five classes, and compared for model fit. There is no single index recommended to identify the most statistically parsimonious solution, so a range of fit indices (Akaike’s Information Criterion (AIC); Bayesian Information Criterion (BIC); Consistent Akaike’s Information Criterion (CAIC), and approximate weight of evidence (AWE); and likelihood tests [17]) are reported to select the most appropriate model for the survey data from a statistical perspective. Data from the interviews were not incorporated into the LCA as the information could not be coded simply into quantitative data that would be informative to the model and interview data were available for a sub-set of services only.

The study was designed a priori with a minimum sample size of 150 services to provide approximately 90% power (based on the bootstrap likelihood ratio test with an alpha of 0.05), or at least 93% power (based on using information criterion), for selecting a three-class model over a two-class model [18]. LCAs were conducted using Mplus, SEM software, version 8.6 [19], to estimate the probability of “service type” membership, given the observed variables. Data wrangling, summary statistics, and plots were conducted using the statistical software, R (version 4.0.3 – 2020-10-10) [20], and making use of packages: *MplusAutomation* [21], *tidyverse* [22], and *psych* [23]. All LCAs used full-information maximum likelihood to deal with any missing data (Missingness summary, Supplementary files, section "Methods", Figure S1).

#### Descriptive analysis

Planned descriptive analysis was undertaken to provide additional context and establish a more detailed framework for service models. No pre-existing framework for this descriptive analysis of service models for children with intellectual disability who display behaviours that challenge was available to use in the current study. Thus, a pragmatic process was used that included nine stages of review by the research team, lead investigator (last author), Family Carer Advisory Group (recruited and supported by the PPI partner organisation), Professional Advisory Group, Study Management Group, and Study Steering Committee; all of whom confirmed the face validity of the groupings of service models within the framework. A detailed outline of the process can be found as supplementary file, section "Results". The descriptive analysis was not at this stage linked to the LCA work, although the research team were not blind to the LCA findings.

##### Statistical analysis of the descriptive framework

Following establishment of the descriptive classifications of services, statistical analysis was conducted to establish whether any differences were present between the groups of services. The purpose of the quantitative analysis was to describe how these service groups differed or were similar across the survey items and to examine whether the service model profiles were distinct from each other. All analyses were conducted using R (version 4.0.3 - 2020-10-10). Continuous survey variables were compared across groups using a one-way ANOVA with Tukey post-hoc tests to show pairwise group differences. Categorical survey variables were compared across groups using Chi square tests of independence, followed by Fisher’s exact approach for post hoc analysis of a chi-squared test [24].

## Results

### Characteristics of services

161 services completed the survey and 60 of those also completed an interview. A complete table of results from the survey is presented in the Supplementary file (Table S1). Table S1 presents the survey item responses as frequency counts and percentages; or means, standard deviations (SDs); medians, and interquartile ranges (IQRs). We report some key characteristics of the services in this section as context for the description of service models.

Services had been in place for an average of 11.89 years (SD=8.67 years), and of the 38 services established for ≤5 years, seven were a temporary or short-term service with a fixed end date. Most services were part of another service (*N=*114, 71.25%) rather than a stand-alone service (*N=*46, 28.75%). The median number of new and re-referrals received by services in a typical year was 75 (IQR=120) and the average proportion of those referred who also displayed behaviours that challenge was 74.54% (SD=46.84). Similarly, the average number of children with intellectual disability who displayed behaviours that challenge currently on a waiting list was 18.69 (SD=31.98). The median current total active service caseload was 61 children who displayed behaviours that challenge (IQR=92). Regarding training, 79.25% (*N=*126) services reported that their staff had specialist training and qualifications in behaviours that challenge (e.g., in Positive Behavioural Support [PBS], or postgraduate specific courses in intellectual disability/autism) beyond their professional training.

In a typical year, the median percentage of accepted referrals for children with intellectual disability who displayed behaviours that challenge where the family did not speak English as a first language was 10 (IQR=15, however, 53% of the data were missing). Very few services made no provision for these families (*n=*2); most provided translated information (*N=*60, 62.5%) and/or interpreter services (*N=*94, 97.92%). Nine services made other provision which typically involved one parent translating for the other, or employing staff who spoke other languages. Services were located across all NHS regions in England (see Table 1).

**Table 1:** Total number of services in each NHS region

**NHS England Region**	**Total number of services**
London	30
North-East and Yorkshire	29
North-West	27
Midlands	23
South-East	21
South-West	17
East of England	14

### Latent class analysis

Five latent class models were initially fitted to the survey data (1-5 class models). From the survey, data coded from 19 items were included in the first set of models fitted. Statistical models with up to four classes were fitted to the data without substantial model fit or convergence issues, but the 5-class model would not converge. For the 5-class LCA model, the model was overly complex to support the data, and so was omitted from the results. A further five of the coded survey items were removed as they were found to be uninformative to the model and potentially reduced model fit. These categorical variables typically had a very narrow range of responses, often almost all services falling into the same category, which when translated into the latent class analyses offered no additional distinction of groups within all services. Details of the variable selection process are documented in supplementary file, Section "Methods".

The resulting final LCA model suggested there were two service models: a specialised service model (Group 1: 31 services [19.65%]) and a broader focus group (Group 2: 130 services [80.35%]). The model fit indices were relatively conclusive indicating support for the 2-class model. Table 2 shows the fit indices and likelihood ratio test results for the various class models. The information criterion (BIC and CAIC) show that the 2-class model is favoured (lowest value is preferred). Similarly, the AWE and both likelihood ratio tests also favoured the 2-class model as the tests showed a non-significant difference was observed between the 2 and 3 class models, indicating little or no improvement of the 3-class model over the 2-class model.

**Table 2:** Model fit indices and likelihood ratio tests

Classes	Parameters	LL^a^	BIC^b^	CAIC^c^	AWE^d^	VLMR_p^e^	LMR_p^f^
1	17	-2011.73	4109.85	4126.85	4135.35		
2	33	-1930.11	4027.90	4060.90	4077.40	<0.01	<0.01
3	49	-1899.49	4047.97	4096.97	4121.47	.65	.65
4	65	-1880.55	4091.38	4156.38	4188.88	>.99	>.99

^a^*LL* loglikelihood

^b^*BIC* Bayes Information Criterion

^c^*CAIC* consistent Akaike information criterion

^d^*AWE* Approximate weight of evidence

^e^*VLMR* Vuong-Lo-Mendell-Rubin likelihood ratio test (*p* value)

^f^*lame* Lo-Mendell-Rubin likelihood ratio test (*p* value)

**Fig. 2:**
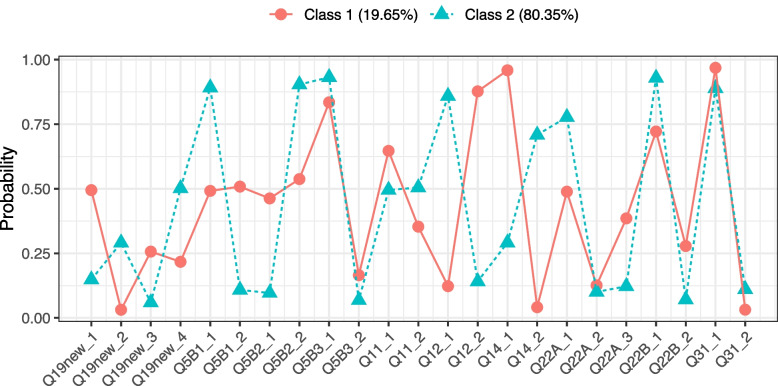
Probability plot of the two-class model. Question (Q5, Q11, Q12, Q14, Q19, Q22, Q31) indexes/coding can be found in supplementary file, section "Discussion", Table S2

Figure 2 shows a graphic representation of the two-class model. The x-axis shows the categorical indicator variables derived from the survey, and the values on the y-axis are the average probability of response to the indicator if with a certain class. For each categorical or binary item, the average probability of response to each category within each class is plotted. For example, Question 19 has four categories of response, so eight probabilities are plotted (four per each class). Table 3 presents the summary of defining features of the LCA defined service models.

**Table 3:** Defining features of the LCA defined service models

**Service Model 1 (Group 1 – specialised service)**	**Service Model 2 (Group 2 – broader focus service)**
51% of services had more than 50% of staff who had training in Positive Behaviour support, and 29% had additional Challenging behaviour training.	22.3% of services had more than 50% of staff who had training in Positive Behaviour support and only 6.9% had additional Challenging behaviour training.
48.4 % had some local authority commissioning (either education or social care or both); 51.6% had some Clinical Commissioning Group (CCG); and 16.1% had some Transforming care Partnership/Integrated Care Systems/ Provider collaborative funding.	90.7% of services had some Clinical Commissioning Group, 10.9% had local authority (either education or social care or both) funding; and 7% had some Transforming Care Partnership/Integrated Care Systems/ Provider collaborative funding
87.7% services were only for children and young people with behaviours that challenge (even if they also have other support needs)	14% services were only for children and young people with behaviours that challenge (even if they also have other support needs)
Mostly professional referral route only with only 3.2% accepting self-referrals.	Most services accepted both professional and self-referral (70.8% of services accepted self-referrals).
On average, the current caseloads was 11.	On average, the current caseloads services was 78.
The average time that services had been established was approximately 7 years.	The average time that services had been established was approximately 13 years.

The LCA supports the general notion of a more specialist behaviours that challenge service model vs. other models of service delivery that include behaviours that challenge pathways. However, the statistical analysis did not sufficiently differentiate the underlying heterogeneity in the generic, broader scope service group. The planned descriptive analysis provided an opportunity to explore these distinctions, identifying further potential service models beyond the statistical analysis.

### Descriptive analysis

The descriptive analysis process led to the development of five service models (Table 4). The specialist behaviours that challenge service model (*BtCSpecialist*) was retained and refined from that identified in the statistical analysis. This service model accounts for the majority of specialist behaviours that challenge services (*n=*27). The largest service model group were generic CAMHS services (*GenCAMHS*), which offered a distinct support pathway for children with intellectual disability who displayed behaviours that challenge, whilst also supporting other children (*n=*69). A related service model, intellectual disability CAMHS (*IDCAMHS*), includes services that identify as stand-alone “ID CAMHS” and provide supports specifically for children with intellectual disability or a broader group of children with neurodevelopmental conditions (*n=*28) with a behaviours that challenge pathway. The smallest service model group describes ‘all-age’ services (*AllAge*; i.e., those crossing ages from early years to adulthood or adolescence to adulthood; *n=*12). These services had some characteristics of other service models but were distinguished as separate because of their broader age coverage which makes them distinct to other service models. Consultation with the study management group and Family Carer Advisory Group indicated a preference to retain this group of services separately given challenges and disruption of transition between services that are commonly experienced. The final service model includes services that focus on children with intellectual disability among other children with disability (*ChildDis*) and offer support for behaviours that challenge in addition to other related services.

**Table 4:** Summary of service models from the descriptive analysis

Service Model	Number of services	Description
*Model 1*: Child and Adolescent Mental Health Services (CAMHS) (G*enCAMHS*)	69	Generic CAMHS that have a distinct treatment pathway to support children aged 0-17 years with ID and BtC, whilst also supporting other children. Services within this model identify as an ID and/or neurodevelopmental pathway, with some describing themselves as offering PBS for BtC either as a separate pathway or as a framework for support. These pathways also typically include support for mental health problems in children with ID/neurodevelopmental conditions.
*Model 2:* Intellectual Disability CAMHS (*IDCAMHS*)	28	Services that identify as stand-alone “ID CAMHS” and provide supports specifically for children with ID. The element of these services being ‘stand-alone’ rather than a distinct pathway within a wider CAMHS is the key factor that makes the model distinct to Model 1 (*GenCAMHS*). Two services that are part of a wider national and specialist CAMHS have been included within this model as they appear to be stand-alone services to support children with ID that also provide/are involved in local services.
*Model 3*: Children and Young People Disability with ID Expertise (*ChildDis*)	25	Services which focus on children with ID/ children with disability, and where supports for BtC are included in addition to other supports. Some services included within this model are community ID nursing teams, where supports offered include assessment and intervention for BtC, whilst the services also offer supports for other needs of children with ID (i.e., sleep, continence, diet/eating, physical and general health).
*Model 4:* Specialist BtC services (*BtCSpecialist*)	27	Services that provide intensive, or specialist supports for BtC, often using PBS. Some of these services are third sector, private services (commissioned to provide PBS supports), and stand-alone specialist children’s services where the focus is BtC. Services also included in this model are intensive/specialist BtC or PBS services for ID that may also have an administrative link to CAMHS, or joint intensive/specialist initiatives between CAMHS and local authority.
*Model 5:* Children and/or adolescents and adult services (*AllAge*)	12	Services which support children as well as adults with ID, or provide specialist/intensive support for children and/or adolescents and adults with ID. They share is a wider/all age approach rather than necessarily being BtC specialist services. Most services in this model are general ID services (dealing with a range of needs, not just BtC), with the same higher level service management structure, yet supports are provided as two teams/specialisms within the same service as there is also a recognised need for differences in staff specialism between adults and children with ID. Some of these services are from a particular region in England which adopts an all-age ID service model approach.

### Cross tabulation of latent class analysis and descriptive analysis findings

A cross tabulation of the service models produced within the LCA and descriptive analysis showed that more specialist behaviours that challenge service model established in the descriptive analysis aligned with Group 1 (specialist model) from the LCA (Table 5) and that the larger broader focus group mainly was broken down into four distinct service models.

**Table 5:** Cross tabulation of the latent class analysis and descriptive analysis findings

**Descriptive classifications**	**Statistical classification**	
	Service Model 1	Service Model 2
G*enCAMHS*	2	67
*IDCAMHS*	2	26
*ChildDis*	2	23
*BtCSpecialist*	23	4
*AllAge*	2	10

#### Statistical comparison across descriptive groups

After defining the descriptive groupings, we analysed them to investigate whether specific differences among the service models were present. On average, the *ChildDis* and *IDCAMHS* services have been established the longest, whereas the *BtCSpecialist* group have been established for the shortest time [F(4, 135)=11.27, *p<*0.001]. *GenCAMHS*, *IDCAMHS* and *ChildDis services* were generally commissioned by a single CCG (75-90% of services) with only a small proportion receiving commissioning from elsewhere (14.3% *IDCAMHS* commissioned in part by Transforming Care Partnership / Integrated Care System/provider collaboratives and 37.5%*ChildDis* commissioned in part by local authorities). *BtCSpecialist* services are most likely to have multiple commissioners (32% of services), with the largest subgroup commissioned by a local authority (52% of services) and CCGs (60% of services). The *AllAge* services were mainly commissioned by a CCG (85.7% of services), although some were commissioned through other structures including NHS England specialist commissioning (7.14%).

The percentage of services within the service models that were for children and young people between the ages 12-25+ ranged from 85.7 -100%, and only a few services provided services from birth (14.3% of *AllAge*; 4% of *ChildDis*; and 1.4% of *GenCAMHS* services). Some specific differences were found between *AllAge* and *GenCAMHS* services for transition age [χ^2^ (4)=17.572, *p=*0.002]: *GenCAMHS* are less likely to have a later age for transition to another service or continuation onto another pathway within the same service. A relationship was observed between service model and whether they offered services to children with intellectual disability and/or autistic children (where autistic children with intellectual disability were also included) only or whether other individuals were also provided for, [χ^2^ (4)=25.937, *p<*0.001]. Posthoc tests revealed that the *BtCSpecialist* service model was equally likely to support both children with an intellectual disability and/or autistic children and other children too. However, the *AllAge* service group was mostly for those with an intellectual disability and/or autism only, whereas the *ChildDis* service group was more likely to include support for other children in addition to those with intellectual disability and those who are autistic.

Compared to other service models, *BtCspecialist* services were more likely to provide support only to children whose behaviour is challenging, and *GenCAMHS* services were less likely to have this specific focus (χ^2^ (4)=55.175, *p<*0.001). Differences in referral pathways (either professional only or professional and self-referral) were observed in *BtCSpecialist* services that were more likely to only accept professional referral, and *GenCAMHS* who were more likely to accept self-referrals too [χ^2^ (4)=23.683, *p<*0.001]. Active caseloads were generally similar across groups but some differences were present [F(4, 134)=21.97, *p<*0.00001; log transformed due to skew in distribution]. *BtCSpecialist* groups having the lowest caseloads and *IDCAMHS* having the largest. Statistically significant differences were found between: *BtCSpecialist* versus *IDCAMHS, GenCAMHS* and *ChildDis* [ all *p<*0.001], and between *AllAge* versus *ChildDis, GenCAMHS, IDCAMHS* [*p=*0.002,*p=*0.002; *p<*0.001respectively].

Proportions of professional groups across the service groups differed [χ^2^ (12)=51.818, *p<*0.001], with specific differences found in all groups except *IDCAMHS*. For services that contained more than 50% of staff in specific roles: *AllAge* staff were mostly learning disability nurses; *BtCSpecialist staff* were mostly psychologists and none had over 50% learning disability nurses; *ChildDis* services did not have any with 50% of staff that were psychologists. Regarding percentage of staff with specialist training in Positive Behaviour Support and behaviours that challenge, service models showed differences for both training types; Positive Behaviour Support [χ^2^ (8)=19.727, *p=*0.011] and challenging behaviour training [χ^2^ (4)=13.459, *p=*0.009]. *BtCSpecialist* services had more services than expected in each of the categories: “no staff or less than 50% with Positive Behaviour Support training,” and “more than 75% of staff have training in Positive Behaviour Support.” For behaviours that challenge training, some differences were present [χ^2^ (4)=13.459, *p=*0.009], but only for the *BtCSpecialist* group as they were more likely to contain more services with over 50% of staff with behaviours that challenge training.

Some differences were found regarding the number of referrals received to the service models [F(4, 136)=10.61, *p<*0.001; log transformed]. *BtCSpecialist* had the lowest number of referrals and *ChildDis* received the highest number of referrals. Specifically, differences were found between the *BtCSpecialist* group and all other services [*AllAge*, *p=*0.003; *ChildDis*, *p<*0.001; *GenCAMHS*, *p<*0.001; *LDCAMHS*, *p<*0.001] with BtC referrals much lower than all other services. We also observed differences between *GenCAMHS* and *ChildDis* as *GenCAMHS* had lower average referrals [*p=*0.018]. Finally, differences in outcome domains used by services to measures children and young people with behaviours that challenge were present [χ^2^ (4)=12.25, *p=*0.016], but this was mainly driven by *GenCAMHS* services that had more services that expected that only used child measures.

## Discussion

We used two planned complementary approaches to determine a typology of ‘service models’ that grouped together similar community services for children with intellectual disabilities who display behaviours that challenge in England. The first approach employed a data driven process (Latent Class Analysis) and found two specific service models could be gleaned from the quantitative information collected from a survey. The second complementary approach used descriptive analysis to incorporate a broader set of information from the survey and in some cases interview data were also used to inform final classifications, leading to identification of five services models. Comparison of the identified service models from each approach indicated that both methods agree on the identification of a specialist behaviours that challenge service model with very close agreement on the services identified under this model. The remaining four service models identified in the descriptive approach were mainly from the statistical method’s “broader focus” group of services.

Given the numbers of services within each of the five service models described, specialist services for children who display behaviours that challenge amongst children may be less common across England than adult services (Hassiotis et al. [14, 15]). If families or referring professionals are looking for specialist behaviours that challenge services, these may be hard to find. It is not altogether clear on name alone that needs related to behaviours that challenge would, for example, be supported via a mainstream CAMHS as there is no reference to behaviours that challenge in the names of most services. Similarly, only a small number of services (7.5%) identified by our study offered an ‘all age’ framework to their service that may support smoother transition into adulthood services and support. Hassiotis et al. [14] did indicate that 16% of the intensive support teams that they identified in adult services accepted referrals for young people in transition (14- to 17-years-old) and 3% indicated that they operate as an all age service perhaps suggesting that this model may be under-represented in our sample (which was recruited from a child services perspective in the main).

Identification of the typology of service models and mapping services within a geographical region or country may have practical benefits for service planning, commissioning and facilitating additional research. In terms of immediate utility, young people, their families and the public would be empowered to identify and consider accessing to specialist services in their local area. Data from the current research may also facilitate policy impact. For example, data could be used to understand whether recommendations from national guidance (such as NICE) are being implemented across England – do the services mapped and models identified match with what would be expected from guidance? Repeated mapping exercises of the sort conducted for this research could also assist in monitoring the implementation of policy similar to the NHS Getting it Right First Time (GIRFT) programme. Advocacy organisations could also use the detailed information from the current study to examine the local availability of services; and potentially challenge commissioners if there are gaps in service scope and coverage.

Considering use in future research, identifying different service models as a typology is a first step in building evidence about the best provision of services for children with intellectual disabilities who display behaviours that challenge. For example, different ways of delivering services may lead to variation in outcomes for children with intellectual disabilities and their families or variation in families’ experiences of receiving supports. If there are certain service models that deliver better outcomes and a better experience for children and families, these may be a priority for commissioning. Similarly, a detailed understanding of what different services models cost to deliver is important both in considering the balance of cost and effectiveness and also setting budgets. Such research may help to inform the design and co-design of services for young people and their family carers. A second stage of our research will investigate these questions in relation to the five service models for children with intellectual disabilities and behaviours that challenge identified in the current study.

## Strengths and limitations

Through the planned use of statistical and descriptive methods, we were able to identify a typology for service models that extended the detail obtained from a statistical approach alone. A similar combination of methods may be useful in other research on defining models of health and care services delivery. A large sample of services from across England was also included, capturing a balance of demographic and geographic factors improving the representativeness of our findings to England.

Despite our best intentions, the statistical analysis had limitations on the amount of information that could be sensibly incorporated for some study variables. Similarly, the sample size was sufficient to test between different class models, but a larger sample may have given additional ability to discriminate more subgroups that was possible in the present study through the planned use of an additional descriptive analysis approach. We had initially estimated the presence of 200-220 eligible services in England based on expert clinician knowledge about services in a sample of local authority areas. Thus, there may not have been a much larger sample of services available. In the study by Hassiotis et al. [14] on adult services, 73 services were included in the analysis. There may be a limited sample size available for similar services research in future in the intellectual disabilities field. It might, therefore, be crucial to include descriptive methods alongside statistical approaches to ensure valid classifications of service models are derived. To add robustness to the method, the research team sought consultation from multiple advisory groups containing professionals (academics, practitioners) and family carers to confirm the face validity of the service models typology.

## Conclusions

A typology of five service models has been described for community health and care services for children with intellectual disabilities who display behaviours that challenge. The most common service model is generic CAMHS services that offer both a distinct treatment pathway to support children with intellectual disability who display behaviours that challenge, whilst also providing support for other children with mental health needs. A related service model is intellectual disability CAMHS that provide supports specifically for children with intellectual disability. These two models constitute 60% of the identified services. The third broader service model focuses on children with disability more generally, where support for behaviours that challenge is included in addition to other services. The smallest service model group offers services to individuals across a wider age range (birth to adulthood, or from 5 years to adulthood). The final service model offers more specialist support with intensive, or specialist support for behaviours that challenge only, often using PBS. These services include some third sector, private services (commissioned to provide PBS supports), and stand-alone specialist children’s services where the focus is behaviours that challenge; and represent approximately 17% of services.

### Supplementary Information

**Additional file 1: Table S1.** Characteristics of services (multiple choice items). **Figure S2.** Counts of responses present within each class in those with responses and missing data. **Figure S3.** presents the patterns of response for Question 3. **Figure S4.** shows the pattern of responses for question 5A. **Figure S5.** shows the pattern of responses to question 5B (part 4). **Figure S6.** presents the patterns of responses for Question 8. **Figure S7.** presents the patterns of responses for Question 9. **Figure S8.** shows the distribution of responses for question 10. **Figure S9.** shows the distribution of responses for question 15. **Figure S10.** shows the pattern of responses for Question 24. **Table S2.** Items for LCA analysis.

## Data Availability

Anonymised data for the LCA analysis and R analysis code can be found on the Open Science Framework repository https://osf.io/8e24a/. The datasets analysed during the current study available from the corresponding author on reasonable request.

## Abbreviations

AIC: Akaike’s Information Criteria
AllAge: Children and/or adolescents and adult services
ANOVA: Analysis of Variance
AWE: approximate weight of evidence
AllAge: Children and/or adolescents and adult services
BIC: Bayes Information Criteria
BtC: Behaviours that Challenge
BtCSpecialist: Specialist services for children with behaviours that challenge
CAIC: Consistent Akaike’s Information Criterion
CAMHS: Child and Adolescent Mental Health Services
CCG: Clinical Commissioning Group
ChildDis: Children and Young People Disability with ID expertise service
FOI: Freedom of Information
ICS: Integrated Care System
IDCAMHS: Intellectual Disability CAMHS
IQR: Interquartile range
IST: Intensive Support Teams
LCA: Latent Class Analysis
NHS: National Health Service
NICE: National Institute for Health and Care Excellence
PBS: Positive Behavioural Support
PPI: Patient and public involvement group
SEM: Structural Equation Modelling
STP: Local authority (education or social care)
