# Supplementary material for: Mapping and identifying service models for community-based services for children with intellectual disabilities and behaviours that challenge in England
Source: BMC Health Serv Res. 2023 Dec 4;23:1354. doi: 10.1186/s12913-023-10388-9 (PMC10696826; doi:10.1186/s12913-023-10388-9)
Supplement: Supplementary file 1 — Additional file 1: Table S1. Characteristics of services (multiple choice items). Figure S2. Counts of responses present within each class in those with responses and missing data. Figure S3. presents the patterns of response for Question 3. Figure S4. shows the pattern of responses for question 5A. Figure S5. shows the pattern of responses to question 5B (part 4). Figure S6. presents the patterns of responses for Question 8. Figure S7. presents the patterns of responses for Question 9. Figure S8. shows the distribution of responses for question 10. Figure S9. shows the distribution of responses for question 15. Figure S10. shows the pattern of responses for Question 24. Table S2. Items for LCA analysis. [file 12913_2023_10388_MOESM1_ESM.docx]

**Supplementary file 1: Mapping and identifying service models for community-based services for children with intellectual disabilities and behaviours that challenge in England**

Emma L. Taylor, Paul A. Thompson, Nicholas Manktelow, Samantha Flynn, David Gillespie, Jill Bradshaw, Nick Gore, Ashley Liew, Mark Lovell, Kate Sutton, Caroline Richards, Stavros Petrou, Peter E. Langdon, Gemma Grant, Vivien Cooper, Kate Seers & Richard P. Hastings

# Section 1 Characteristics of Services (Survey item summaries)

Table S1: Characteristics of services (multiple choice items)

| **Question** | **Response Category** | **Summary statistics** | |  |  |
| --- | --- | --- | --- | --- | --- |
| Q1 | **How long has the service been in place?** | ***mean*** | ***SD*** | ***median*** | ***IQR*** |
|  | years established | 11.89 | 8.67 | 10.167 | 11.8125 |
|  |  |  |  |  |  |
| Q2 | **Is the service a temporary or short term service/project with a fixed end date?** | ***N*** | ***%*** |  |  |
|  | Not a temporary/ short term service (No) | 31 | 81.6 |  |  |
|  | short term service (yes) | 7 | 18.4 |  |  |
|  |  |  |  |  |  |
| Q3 | **Does the service stand-alone or is it part of/a sub-team of another service?** | ***N*** | ***%*** |  |  |
|  | Part of another service | 114 | 71.25 |  |  |
|  | Stand alone | 46 | 28.75 |  |  |
|  |  |  |  |  |  |
| Q5 | **Who commissions the service? If this service is jointly commissioned, please select all that apply. If there is more than one commissioner, you will then be asked to explain the rough proportions of share in funding.** | ***N*** | ***%*** |  |  |
|  | Clinical Commissioning Group | 133 | 81.125 |  |  |
|  | Local Authority: Social Care | 27 | 16.875 |  |  |
|  | NHS England Specialist Commissioning | 12 | 7.5 |  |  |
|  | Other | 11 | 4.375 |  |  |
|  | Local Authority: Education | 8 | 5 |  |  |
|  | Transforming Care Partnership | 8 | 5 |  |  |
|  | Integrated Care System (ICS) | 5 | 3.125 |  |  |
|  | Private individual | 1 | 0.625 |  |  |
|  | Sustainable Transformation Partnership (STP) | 1 | 0.625 |  |  |
|  | Private organisation or company | 0 | 0 |  |  |
|  | Voluntary organisation or charity | 0 | 0 |  |  |
|  |  |  |  |  |  |
| Q6 | **You selected more than one commissioner. Please tell us the approximate percentage of funding that comes from each of the options you selected? *(only display if 2 or more options selected in Q5. Only options selected in Q5 displayed)*** | ***mean*** | ***sd*** |  |  |
|  | Private organisation or company | - | - |  |  |
|  | Voluntary organisation or charity | - | - |  |  |
|  | Local Authority: Social Care | 40.28 | 32.83 |  |  |
|  | Private individual | 37.25 | 39.72 |  |  |
|  | Sustainable Transformation Partnership (STP) | 28.33 | 44.91 |  |  |
|  | NHS England Specialist Commissioning | 19.29 | 30.61 |  |  |
|  | Local Authority: Education | 18.33 | 19.14 |  |  |
|  | Other | 10 | - | (single response) | |
|  | Clinical Commissioning Group | 1.4 | 3.13 |  |  |
|  | Transforming Care Partnership | 0 | 0 |  |  |
|  | Integrated Care System (ICS) | 0 | 0 |  |  |
|  |  |  |  |  |  |
| Q8 | **What are the ages of the children and young people with behaviours that challenge who can access the service? Please select all that apply** | ***N*** | ***%*** |  |  |
|  | 16-19 years | 159 | 98.76 |  |  |
|  | 12-15 years | 157 | 97.52 |  |  |
|  | 5-11 years | 156 | 96.89 |  |  |
|  | 0-4 years | 118 | 73.29 |  |  |
|  | 20-25 years | 13 | 8.08 |  |  |
|  | 25+ years | 13 | 8.08 |  |  |
|  |  |  |  |  |  |
| Q9 | **At what age do children and young people with behaviours that challenge transition out of the service to a service for individuals who are older?** | ***N*** | ***%*** |  |  |
|  | 18 years of age | 130 | 81.76 |  |  |
|  | There is no adult service to which they transition | 9 | 5.66 |  |  |
|  | 19 years of age | 7 | 4.4 |  |  |
|  | They stay with the same service/team | 5 | 3.15 |  |  |
|  | 17 years of age | 3 | 1.87 |  |  |
|  | 25 years of age | 2 | 1.26 |  |  |
|  | 20 years of age | 1 | 0.63 |  |  |
|  | 24 years of age | 1 | 0.63 |  |  |
|  | 16 years of age | 0 | 0 |  |  |
|  | 21 years of age | 0 | 0 |  |  |
|  | 22 years of age | 0 | 0 |  |  |
|  | 23 years of age | 0 | 0 |  |  |
|  | 26+ years of age | 0 | 0 |  |  |
|  |  |  |  |  |  |
| Q10 | **For those children and young people with behaviours that challenge transitioning out of the service, to what service(s) do they transition? Please select all that apply *(not displayed if ‘there is no adult service to which they transition’ is selected in Q9)*** | ***N*** | ***%*** |  |  |
|  | Community adult learning disability services | 139 | 95.86 |  |  |
|  | Adult Intensive Support Team (IST) | 56 | 38.62 |  |  |
|  | Other | 46 | 31.72 |  |  |
|  | Community forensic services | 19 | 13.1 |  |  |
|  |  |  |  |  |  |
| Q11 | **What groups of children and young people with behaviours that challenge is the service for (in terms of inclusion criteria for the service)? Please select all that apply** | ***N*** | ***%*** |  |  |
|  | Children and young people with Learning (intellectual) disabilities | 158 | 98.14 |  |  |
|  | Children and young people who both have learning (intellectual) disabilities and who are autistic | 150 | 93.17 |  |  |
|  | Children with global developmental delay | 113 | 70.19 |  |  |
|  | Children and young people who do not have a learning (intellectual) disability | 54 | 33.54 |  |  |
|  | Other children and young people with particular 'diagnosis' | 50 | 31.06 |  |  |
|  | Other diabled children and young people | 43 | 26.71 |  |  |
|  | Non-disabled children and young people | 11 | 6.83 |  |  |
|  |  |  |  |  |  |
| Q12 | **Is the service only for children and young people with behaviours that challenge (even if they also have other support needs)?** | ***N*** | ***%*** |  |  |
|  | No | 115 | 71.43 |  |  |
|  | Yes | 46 | 28.57 |  |  |
|  |  |  |  |  |  |
| Q14 | **Who can refer children and young people with behaviours that challenge into your service? Please select all that apply** | ***N*** | ***%*** |  |  |
|  | Social services | 149 | 92.55 |  |  |
|  | Other health professionals | 141 | 87.58 |  |  |
|  | Paediatricians | 138 | 85.71 |  |  |
|  | Child and Adolescent Mental Health Services (CAMHS) | 137 | 85.09 |  |  |
|  | School - Special | 134 | 83.23 |  |  |
|  | General Practitioners/Primary Care | 132 | 81.99 |  |  |
|  | School - mainstream | 112 | 69.57 |  |  |
|  | Third Sector organisations | 97 | 60.25 |  |  |
|  | Pre-schools | 95 | 59.01 |  |  |
|  | Other | 43 | 26.71 |  |  |
|  | Self-referrals/Referral directly from the child's carer | 0 | 0 |  |  |
|  |  |  |  |  |  |
| Q15 | **In a typical year, how many referrals to the service are received (new and re-referrals)?** | ***Mean*** | ***SD*** | ***Median*** | ***IQR*** |
|  | **Numerical** | 225.09 | 607.5 | 75 | 120 |
|  |  |  |  |  |  |
| Q16 | **Q16 Approximately what proportion of referrals (new and re-referrals) for children and young people with behaviours that challenge are accepted in a typical year?** |  |  |  |  |
|  | proportion of referrals (new and re-referrals) for children and young people with behaviours that challenge | 78.54 | 46.84 | 80 | 21.25 |
|  |  |  |  |  |  |
| Q17 | **Approximately how many children and young people with behaviours that challenge are currently on the waiting list for the service?** |  |  |  |  |
|  | Number on the waiting list | 18.69 | 31.98 | 8 | 23.5 |
|  |  |  |  |  |  |
| Q18 | **Approximately what is the current total active caseload for the service of children and young people with behaviours that challenge?** |  |  |  |  |
|  | Current Active caseload | 104.97 | 171.56 | 61 | 92 |
|  |  |  |  |  |  |
| Q19 | **For each of the following professional groups, please indicate how many staff in the service are from this background. If nobody of this profession works in the service, please answer '0'. Please categorise staff by main role. There will be a follow up question asking about approximate full time equivalent for those professional groups that you indicate you have 1 or more staff of in your service. If there are staff in your service from other backgrounds, please indicate these in the ‘other care staff’ option.** |  |  |  |  |
|  | Average staff (Other care staff) | 70.71 | 643.81 | 1 | 2 |
|  | Average staff (Learning Disability Nurse) | 2.49 | 2.61 | 2 | 4 |
|  | Average staff (Support Worker) | 1.7 | 7.41 | 0 | 2 |
|  | Average staff (Clinical Psychologist) | 1.5 | 1.39 | 1 | 1 |
|  | Average staff (Psychiatrist (e.g., Consultant Psychiatrist, Staff Grade Doctor)) | 0.81 | 0.84 | 1 | 1 |
|  | Average staff (Assistant Psychologist) | 0.66 | 0.95 | 0 | 1 |
|  | Average staff (Social Worker) | 0.61 | 2.54 | 0 | 0 |
|  | Average staff (Occupational Therapist) | 0.54 | 1.24 | 0 | 1 |
|  | Average staff (Mental Health Nurse) | 0.42 | 1.06 | 0 | 0 |
|  | Average staff (Speech and Language Therapist) | 0.34 | 0.63 | 0 | 1 |
|  | Average staff (Helath care assistant) | 0.27 | 0.82 | 0 | 0 |
|  | Average staff (Qualified Teacher) | 0.12 | 0.53 | 0 | 0 |
|  | Average staff (General Nurse) | 0.07 | 0.28 | 0 | 0 |
|  | Average staff (Assistant Social Worker) | 0 | 0 | 0 | 0 |
|  | Average staff (Dietician) | 0 | 0 | 0 | 0 |
|  | Average staff (Physiotherapist) | 0 | 0 | 0 | 0 |
|  |  |  |  |  |  |
| Q20 | **For each of the following professional groups that you indicated has at least 1 member of staff working in your service, please indicate the approximate full time equivalent for each group. *(options only displayed if they were selected in Q19)*** |  |  |  |  |
| (proportions) | Average staff (Speech and Language Therapist) | 15.8 | 107 | 1 | 1.5 |
|  | Average staff (Support Worker) | 4.62 | 14.5 | 1.9 | 1.55 |
|  | Average staff (Clinical Psychologist) | 3.34 | 4.49 | 1 | 3.2 |
|  | Average staff (Mental Health Nurse) | 2.95 | 2.07 | 2.5 | 2.9 |
|  | Average staff (Assistant Psychologist) | 1.98 | 4.32 | 1 | 1.4 |
|  | Average staff (Physiotherapist) | 1.43 | 1.43 | 1 | 0.9 |
|  | Average staff (General Nurse) | 1.29 | 1.81 | 1 | 0.4 |
|  | Average staff (Assistant Social Worker) | 1.12 | 0.564 | 1 | 0 |
|  | Average staff (Occupational Therapist) | 1.1 | 0.897 | 1 | 0.325 |
|  | Average staff (Learning Disability Nurse) | 0.919 | 0.557 | 1 | 0.288 |
|  | Average staff (Helath care assistant) | 0.756 | 0.501 | 0.65 | 0.6 |
|  | Average staff (Other care staff) | 0.744 | 0.543 | 0.6 | 0.6 |
|  | Average staff (Social Worker) | 0.712 | 0.633 | 0.55 | 0.6 |
|  | Average staff (Dietician) | 0 | - | 0 | 0 |
|  | Average staff (Psychiatrist (e.g., Consultant Psychiatrist, Staff Grade Doctor)) | 0 | - | 0 | 0 |
|  | Average staff (Qualified Teacher) | 0 | - | 0 | 0 |
|  |  |  |  |  |  |
| Q21 | **Do any of the staff in the service have any specialist training and qualifications (with some certification such as University or other training provider awards/credits) in behaviours that challenge beyond their professional training? For example, in positive behaviour support?** | ***N*** | ***%*** |  |  |
|  | Yes | 126 | 79.25 |  |  |
|  | No | 33 | 20.75 |  |  |
|  |  |  |  |  |  |
|  |  |  |  |  |  |
| Q22 | **How many staff in the service have had specialist training and qualifications in behaviours that challenge beyond their professional training? Please enter total number of staff for all that apply. If no staff have these qualifications please enter 0 (zero). *(only displayed if ‘yes’ selected in Q21)*** | ***Mean*** | ***SD*** | ***Median*** | ***IQR*** |
|  | Positive behavioural support | 5.38 | 14.6 | 3 | 3.25 |
|  | Other | 3.67 | 5.46 | 1.5 | 4 |
|  | Challenging behaviour course | 3.24 | 9.46 | 1 | 2.5 |
|  |  |  |  |  |  |
| Q23 | **What assessment approaches does your service typically carry out for referrals of children and young people with behaviours that challenge? Please select all that apply.** | ***N*** | ***%*** |  |  |
|  | Functional assessment of challenging behaviour | 150 | 93.17 |  |  |
|  | Mental health assessment | 105 | 65.22 |  |  |
|  | Medicines/drug review | 103 | 63.98 |  |  |
|  | General health screen/assessment | 85 | 52.8 |  |  |
|  | Cognitive assessment | 81 | 50.31 |  |  |
|  | Autism assessment | 68 | 42.24 |  |  |
|  | Sensory assessment | 65 | 40.37 |  |  |
|  | Other | 64 | 39.75 |  |  |
|  | Communications assessment | 57 | 35.4 |  |  |
|  |  |  |  |  |  |
| Q24 | **What intervention approaches does your service typically carry out that involve directly delivering interventions to children and young people with behaviours that challenge? Please select all that apply.** | ***N*** | ***%*** |  |  |
|  | Behavioural interventions that concern behaviours that challenge | 156 | 97.5 |  |  |
|  | Writing a behaviour support plan | 153 | 95.62 |  |  |
|  | Increasing communication skills | 124 | 77.5 |  |  |
|  | Delievering a mulit-element behaviour support plan | 122 | 76.25 |  |  |
|  | Increasing other adaptive skills | 116 | 72.5 |  |  |
|  | Psychological therapies for mental health problems | 111 | 69.38 |  |  |
|  | Psychological / pharmalogical interventions for poor sleep | 104 | 65 |  |  |
|  | Pharmalogical inteventions for mental health problems | 96 | 60 |  |  |
|  | Sensory interventions | 86 | 53.75 |  |  |
|  | Reducing challenging behaviour using medication | 74 | 46.25 |  |  |
|  | Other | 59 | 36.88 |  |  |
|  | Physical health interventions | 44 | 27.5 |  |  |
|  | Other therapies (e.g. art therapies, music therapy, play therapy) | 40 | 25 |  |  |
|  |  |  |  |  |  |
| Q25 | **What supports for children and young people with behaviours that challenge does your service typically use that involve supporting others to deliver interventions (e.g., training)? Please select all that apply** | ***N*** | ***%*** |  |  |
|  | Consulting on individual cases | 142 | 88.2 |  |  |
|  | Providing resources for families to use at home | 134 | 83.23 |  |  |
|  | Mentoring/supervising other staff to deliver a behaviour support plan | 124 | 77.02 |  |  |
|  | Training family carers | 121 | 75.16 |  |  |
|  | Parenting programmes | 104 | 64.6 |  |  |
|  | Training in assessment and intervention for challenging behaviour | 88 | 54.66 |  |  |
|  | Training paid carers | 81 | 50.31 |  |  |
|  | Wellbeing interventions for families | 74 | 45.96 |  |  |
|  | Other | 32 | 19.88 |  |  |
|  |  |  |  |  |  |
| Q26 | **In a typical year, for approximately what percentage of accepted referrals (new and re-referrals) for children and young people with behaviours that challenge does the family not speak English as a first language** | ***Mean*** | ***SD*** | ***Median*** | ***IQR*** |
|  |  | 14.2 | 16.2 | 10 | 15 |
|  |  |  |  |  |  |
| Q27 | **Are language translation services provided in your service for people who do not communicate in English or where English is an additional language? Please select all that apply.** | ***N*** | ***%*** |  |  |
|  | Interpretation services | 94 | 97.92 |  |  |
|  | Translated written information | 60 | 62.5 |  |  |
|  | Other | 9 | 9.38 |  |  |
|  | No language translation services are provided | 2 | 2.08 |  |  |
|  |  |  |  |  |  |
| Q28 | **Are there any vacant (unfilled) posts in the service currently?** | ***N*** | ***%*** |  |  |
|  | Yes (vacant posts) | 62 | 61.39 |  |  |
|  | No | 39 | 38.61 |  |  |
|  |  |  |  |  |  |
| Q29 | **For each of the following professional groups, please indicate how many vacant (unfilled) posts there are in the service from this background. If there are no vacant (unfilled) posts of this profession in the service please answer '0'. Please categorise vacancies by main role. There will be a follow up question asking about approximate full time equivalent for vacant (unfilled) posts in your service. If there are vacant (unfilled) posts in your service for other backgrounds, please indicate these in the ‘other care staff’ option *(only display if ‘yes’ selected in Q28)*** | ***Mean*** | ***SD*** | ***Median*** | ***IQR*** |
|  | Average staff (Other care staff) | 2.21 | 4.3 | 1 | 2 |
|  | Average staff (Support Worker) | 0.926 | 3.84 | 0 | 0 |
|  | Average staff (Clinical Psychologist) | 0.898 | 0.818 | 1 | 1 |
|  | Average staff (Learning Disability Nurse) | 0.826 | 0.654 | 1 | 1 |
|  | Average staff (Assistant Psychologist) | 0.362 | 0.552 | 0 | 1 |
|  | Average staff (Occupational Therapist) | 0.321 | 0.476 | 0 | 1 |
|  | Average staff (Psychiatrist (e.g., Consultant Psychiatrist, Staff Grade Doctor)) | 0.312 | 0.527 | 0 | 1 |
|  | Average staff (Social Worker) | 0.231 | 0.992 | 0 | 0 |
|  | Average staff (Speech and Language Therapist) | 0.197 | 0.375 | 0 | 0 |
|  | Average staff (Mental Health Nurse) | 0.167 | 0.461 | 0 | 0 |
|  | Average staff (Helath care assistant) | 0.0667 | 0.242 | 0 | 0 |
|  | Average staff (Dietician) | 0.0385 | 0.196 | 0 | 0 |
|  | Average staff (Qualified Teacher) | 0.0385 | 0.196 | 0 | 0 |
|  | Average staff (Assistant Social Worker) | 0 | 0 | 0 | 0 |
|  | Average staff (General Nurse) | 0 | 0 | 0 | 0 |
|  | Average staff (Physiotherapist) | 0 | 0 | 0 | 0 |
|  |  |  |  |  |  |
| Q30 | **For each of the following professional groups that you indicated you have at least 1 vacant (unfilled) post in your service, please indicate the approximate total full time equivalent for the vacancies. *(options only displayed if they were selected in Q29)*** | ***Mean*** | ***SD*** | ***Median*** | ***IQR*** |
|  | Average staff (General Nurse) | - | - | - | - |
|  | Average staff (Mental Health Nurse) | - | - | - | - |
|  | Average staff (Qualified Teacher) | - | - | - | - |
|  | Average staff (Other care staff) | 6 | 9.35 | 1.5 | 5.5 |
|  | Average staff (Support Worker) | 3.53 | 5 | 1.2 | 1.25 |
|  | Average staff (Dietician) | 3 | 2.83 | 3 | 2 |
|  | Average staff (Occupational Therapist) | 1.2 | 0.488 | 1 | 0.5 |
|  | Average staff (Physiotherapist) | 1.2 | 0.542 | 1 | 0.3 |
|  | Average staff (Assistant Social Worker) | 1.1 | 0.62 | 1 | 0.2 |
|  | Average staff (Helath care assistant) | 1 | 0 | 1 | 0 |
|  | Average staff (Psychiatrist (e.g., Consultant Psychiatrist, Staff Grade Doctor)) | 1 | - | 1 | 0 |
|  | Average staff (Clinical Psychologist) | 0.973 | 0.352 | 1 | 0 |
|  | Average staff (Assistant Psychologist) | 0.878 | 0.519 | 1 | 0.5 |
|  | Average staff (Learning Disability Nurse) | 0.6 | 0.255 | 0.6 | 0.1 |
|  | Average staff (Social Worker) | 0.6 | - | 0.6 | - |
|  | Average staff (Speech and Language Therapist) | 0.2 | - | 0.2 | 0 |
|  |  |  |  |  |  |
| Q31 | **What outcome domains does your service typically measure for children and young people with behaviours that challenge and their families? Please select all that apply** | ***N*** | ***%*** |  |  |
|  | Family carer experience and satisfaction with services/support received | 139 | 87.97 |  |  |
|  | The child and/or young person's behaviours that challenge | 133 | 84.18 |  |  |
|  | The child and/or young person's mental health (e.g. anxiety, mood/depression) | 119 | 75.32 |  |  |
|  | The child and/or young person's quality of life | 113 | 71.52 |  |  |
|  | Family carer well-being and quality of life (including quality of life of the family as a whole) | 107 | 67.72 |  |  |
|  | The child and/or young person's skills, such as communication skill, social skills independence skills | 86 | 54.43 |  |  |
|  | Other |  |  |  |  |
|  |  |  |  |  |  |
| Q32 | **What methods does the service regularly use to obtain feedback about the service from children and young people with behaviours that challenge and their families and others? Please select all that apply.** | ***N*** | ***%*** |  |  |
|  | Service satisfaction questionnaires with family carers | 149 | 93.71 |  |  |
|  | Service satisfaction questionnaires with children and young people | 107 | 67.3 |  |  |
|  | Interviews with family carers | 65 | 40.88 |  |  |
|  | Professionals or staff from other agencies | 60 | 37.74 |  |  |
|  | Service satisfaction questionnaires with other staff | 57 | 35.85 |  |  |
|  | Interviews with children and young people | 38 | 23.9 |  |  |
|  | Interviews with other staff | 36 | 22.64 |  |  |
|  | Other | 32 | 20.13 |  |  |
|  | Focus groups with children and young people | 16 | 10.06 |  |  |
|  | Focus groups with family carers | 16 | 10.06 |  |  |
|  | Advisory groups with children and young people | 13 | 8.18 |  |  |
|  | Advisory groups with family carers | 12 | 7.55 |  |  |
|  | Focus groups with other staff | 7 | 4.4 |  |  |
|  | Advisory groups with other staff | 3 | 1.89 |  |  |
|  | None | 2 | 1.26 |  |  |
|  |  |  |  |  |  |
| Q33 | **Has the COVID-19 pandemic resulted in substantial changes to the delivery of your services for children and young people with behaviours that challenge and their families? Please select all that apply.** | ***N*** | ***%*** |  |  |
|  | Increase in remote working for staff | 95 | 95 |  |  |
|  | Increase in online completion of assessments | 77 | 77 |  |  |
|  | Delivering more interventions online | 70 | 70 |  |  |
|  | No changes | 0 | 0 |  |  |
|  | Hybrid delivery of services (part online, part face-to-face) | 0 | 0 |  |  |
|  | Adapted interventions (e.g., reduced/increased frequency of sessions or length of interventions) | 0 | 0 |  |  |
|  | Other | 0 | 0 |  |  |

# Section 2 Variable decision process

All variables from the survey are not necessarily be included into the latent class analysis (LCA) as some are not informative to the model but will be used as part of the description of classes and in further discussion around the identification of services.

The LCA analysis can incorporate multiple types of variables including continuous, categorical, and dichotomous items. There are, however, some limitations on those variable types to ensure that modelling assumptions are met, and model fitting issues are avoided or at least minimised in advance.

As part of the variable selection process, data were summarised using descriptive statistics and plots to provide an overview of each question’s responses. Categorical variables were summarised as counts and percentages. Similarly, continuous variables reported means and SDs; alternatively, if skewed or non-normal, median and interquartile range.

## Variables omitted prior to analysis

The following sections provide an overview of the reasoning for omission of particular survey questions from the latent class analysis. Each subsection describes the reasoning for omission according to different criteria when assessing the data for inclusion.

### Questions removed as requested by some service providers

The following questions were removed from the survey at the request of some service providers, so will not be included into the analysis or discussions for mapping services.

- Q7: What is the approximate total annual budget for the service?
- Q29 For each of the following professional groups, please indicate how many vacant (unfilled) posts there are in the service from this background.
- Q30 For each of the following professional groups that you indicated you have at least 1 vacant (unfilled) post in your service, please indicate the approximate total full time equivalent for the vacancies.

### Information is not compatible or not informative for the analysis

Several variables were included into the survey which provided some descriptive information that would be useful to describe the service but would not be informative when distinguishing service model classes in the analysis. These questions were initially screened out following discussions with the CI, Prof. Richard Hastings; Co-I, Dr David Gillespie; and MELD research team at Warwick. The information from these questions were considered likely uninformative for discriminating service models, and inclusion into the model would not likely be beneficial. These variables would still be of interest descriptively. The following variables fall under these criteria:

- Q2: Is the service a temporary or short-term service/project with a fixed end date?
- Q10: For those children and young people with behaviours that challenge transitioning out of the service, to what service(s) do they transition? (linked question to Q9)
- Q26: In a typical year, for approximately what percentage of accepted referrals (new and re-referrals) for children and young people with behaviours that challenge does the family not speak English as a first language?
- Q27 Are language translation services provided in your service for people who do not communicate in English or where English is an additional language?
- Q28 Are there any vacant (unfilled) posts in the service currently?
- Q32 What methods does the service regularly use to obtain feedback about the service from children and young people with behaviours that challenge and their families and others?

### Too much missing information in responses

After the initial screen of variables (6.1), we took a more data-focused approach to screening out variables as potential LCA variables. Summary statistics and missing data information were generated and examined prior to this stage. Variables that were missing substantial amounts of responses would be problematic to include as the amount of missing data in those questions were often substantial. Consequently, use of missing data methods in the analysis would likely be insufficient to sensibly remove bias from results using these variables. Variables omitted from consideration due to missingness included:

- Q15: In a typical year, how many referrals to the service are received (new and re-referrals)?
- Q16: Approximately what proportion of referrals (new and re-referrals) for children and young people with behaviours that challenge are accepted in a typical year?
- Q17: Approximately how many children and young people with behaviours that challenge are currently on the waiting list for the service?

### Patterns of responses are too sparse to be informative

A number of the questions from the survey gave the option to select multiple response categories. For descriptive purposes these questions provide a rich source of information but, from a data analysis perspective, these response patterns can be difficult to manipulate into useable variables for analysis. In questions that were found to have a smaller number of response patterns (i.e. fewer combinations of response categories checked), we were able to reduce this information to a categorical variable (See Appendix in MELD protocol). However, in some questions, the sheer volume of potential response patterns was vast and made condensing this information into sensible categories very difficult. In those questions with mostly sparsely populated response patterns (i.e. patterns with maybe only one or two services responding the same), the research team tried to group similar response patterns to reduce the potential number of categories, but in some cases, there was no sensible way to do this. The following questions were removed from the list to include in the LC analysis :

- Q13: You said that the service is not exclusively for children and young people with behaviours that challenge. Please describe who else/what other groups the service is for. (Linked to Q12, information was from an open-ended response, so research team discussed, but ultimately decided to use for descriptive purposes only).
- Q25 What supports for children and young people with behaviours that challenge does your service typically use that involve supporting others to deliver interventions

### Given responses, meaningful reduction in categories cannot be defined

A final inspection of the potential questions, indicated from discussions with the CI, Prof Hastings, and the research team, that one question’s pattern of responses was not providing meaningful distinction. In this variable, many patterns of responses included two key categories that accounted for the vast majority of service types, so little distinction would be potentially gained from combinations of these variables and other more general items. Meaningful distinction could not be found among the response patterns in the following question:

- Q23: What assessment approaches does your service typically carry out for referrals of children and young people with behaviours that challenge? (Specifically, nearly every service included functional assessment and also nearly all include a range of different assessment approaches, making distinction relatively unmeaningful).

## Variables omitted after initial run of analysis

The following sections provide an overview of the reasoning for omission of particular survey questions from the latent class analysis after an initial run of the analysis. Each subsection describes the reasoning for omission according to different criteria when assessing the data for inclusion.

### Non-informative to the model

After an initial run of the LCA model, the following items were found to be non-informative to the model as they had most responses in one category or all responses fell into one class only which caused model issues.

- Q8: What are the ages of children and young people with behaviours that challenge who can access the service?
- Q9: At what age do children and young people with behaviours that challenge transition out of the service for individuals who are older?
- Q24: What intervention approaches doe your service typically carry out that involve directly delivering interventions to children and young people with behaviours that challenge?
- Q5(4): Who commissions the service? (recategorized into 4 binary response variables for the four most common commissioners -CCG, local authority, TCP/ICS/provider collaborative, NHS specialist). The 4^th^ binary variable (NHS specialist) was dropped very few services responded to this and it was relatively balanced across classes in all models.

### Overly restrictive to the model

After an initial run of the LCA model, the following items were found to be overly restrictive to the model as they strongly dictated how groups formed without consideration of other measures,

- Q3/4: recoded version of: “Does the service stand-alone or part of/a sub-team of another service?” and, “Please describe what other service or sub-team your service is part of?”.

## Omitted variables’ summary statistics by class

Some variables were removed from the survey part way through the data collection as they were found to be uninformative or too much information was missing to be used in the analysis. The remaining were included into the analysis where possible or omitted and used for descriptive purposes in the qualitative evaluation. In this section, summary statistics for these variables are presented as supplementary to the main LCA analysis. Each variable’s summaries are presented per group (service model).

### Question 2: “Is the service a temporary or short term service/project with a fixed end date?”

This item was not included into the analysis as it was linked to question 1 (how long has the service been in place). Only those responding that the service had been running for less than 5 years had values recorded. This meant for the main analysis that a significant proportion of the data were missing to the extent that it was not possible to include without incurring significant bias.

Figures S1 and S2 show the extent of the missing data and the proportions of responses present within each class in those with responses respectively. We see that Question 2 has 76% of the data missing for this variable which is not possible to use imputation or full information maximum likelihood (FIML) sensibly.

Figure S1: Missing data summary


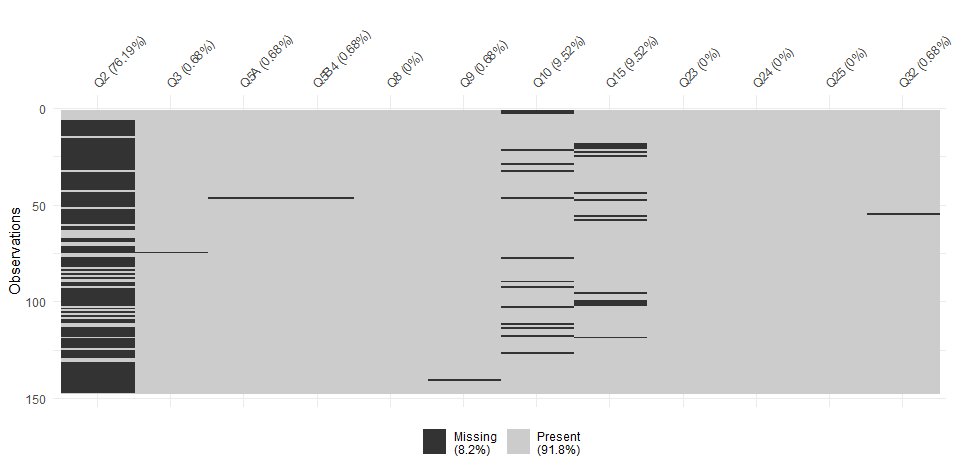


Figure S2: Counts of responses present within each class in those with responses and missing data.


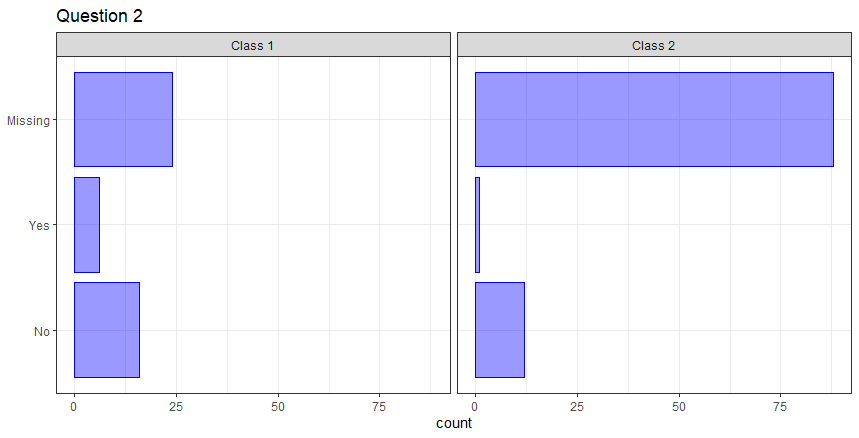


### Question 3|4: Service descriptions (stand alone/part of another service/description)

Question 3 was found to be not informative to the model, so was reconfigured to incorporate information from Question 4. We present the raw information from Question 3 only as question 4 is a free-text response. Figure S3 shows the pattern of responses for this variable split by class (service model). Proportionally, there is little difference in the classes indicating little distinction from this variable.

Figure S3 presents the patterns of response for Question 3.


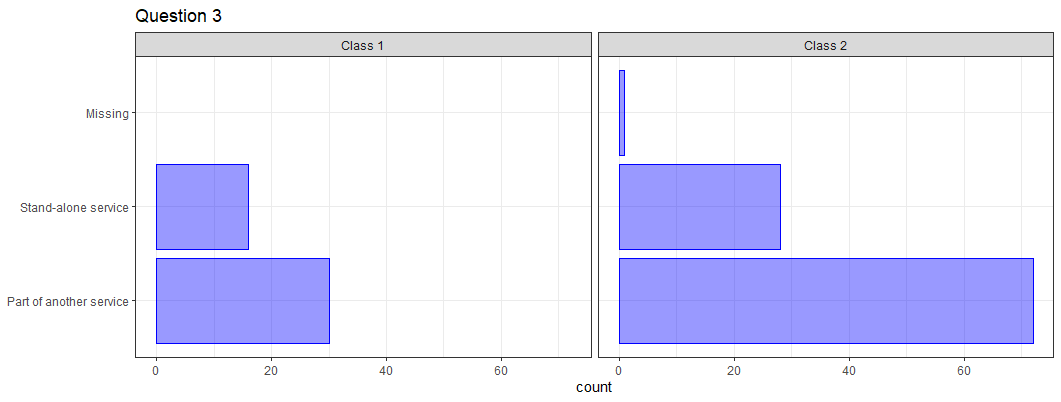


### Question 5A: Single or multiple commissioners for the service?

This question was a recategorization of the original question 5 from the survey. The question was split into parts to make best use of the data. In the first part, it was established whether this was a single or multiple commissioners. The next parts Questions 5B part 1-4 established whether funding was from any of the main commissioners (Local authority: education or social care, Clinical commissioning group; Transforming care partnership/Integrated care systems/provider collaborative; or NHS England specialist commissioning). Again, proportionally there is little difference in the classes indicating little distinction from this variable (Figure S4).

Figure S4 shows the pattern of responses for question 5A.


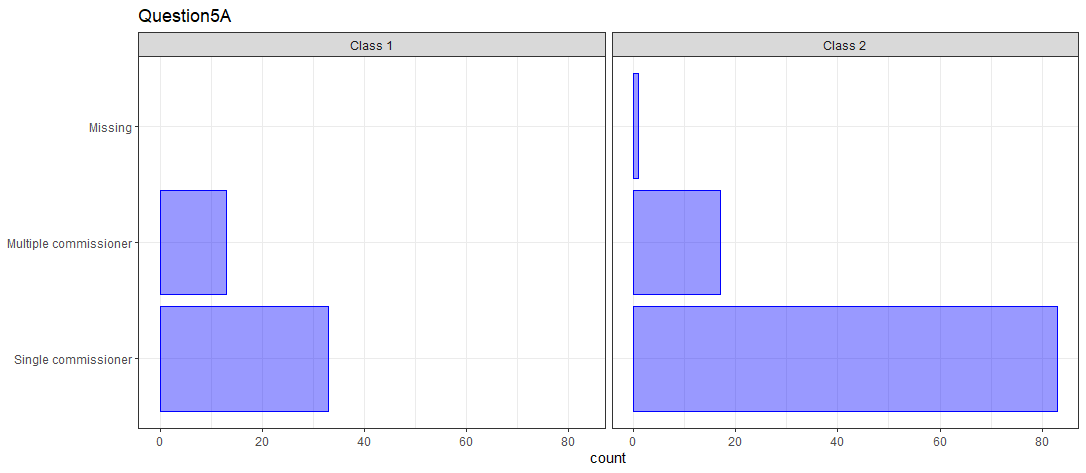


### Question 5B -part 4: NHS England specialist commissioning

This question was a recategorization of the original question 5 from the survey. We took the most commonly responded commissioner types and created several binary response variables from the original data. This was then included in to the model. This binary item was removed from the analysis as it had too few responses in the ‘yes’ category to be useful when determining groups. The number of services that indicated an NHS England specialist commissioner was very similar in both classes, so is unlikely to offer much distinction based on this question (Figure S5).

Figure S5 shows the pattern of responses to question 5B (part 4).


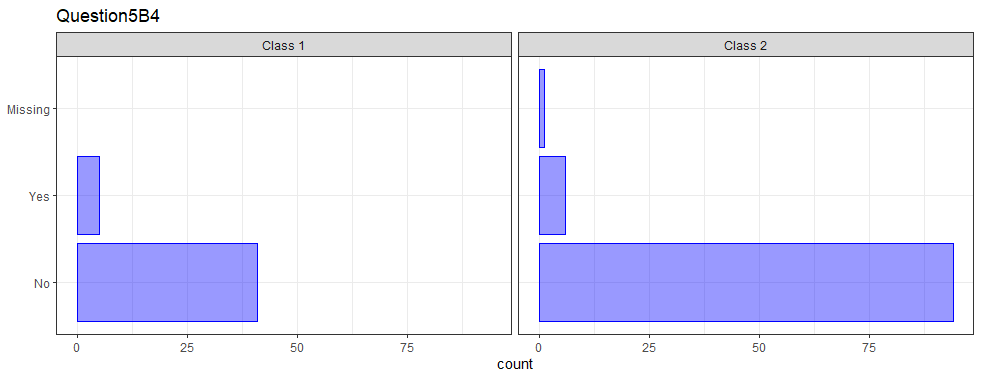


### Question 8: What are the ages of the children and young people with behaviours that challenge who can access the service?

Figure S6 show all patterns of responses from the original survey, i.e. each combination of age ranges checked in the survey. The data were attempted to be recategorized to be more informative to the model, splitting the response patterns into: all age (0-25+) vs adolescent and young adult services (12-25+), but this provided little additional information to the model, so this was removed.

Figure S6 presents the patterns of responses for Question 8.


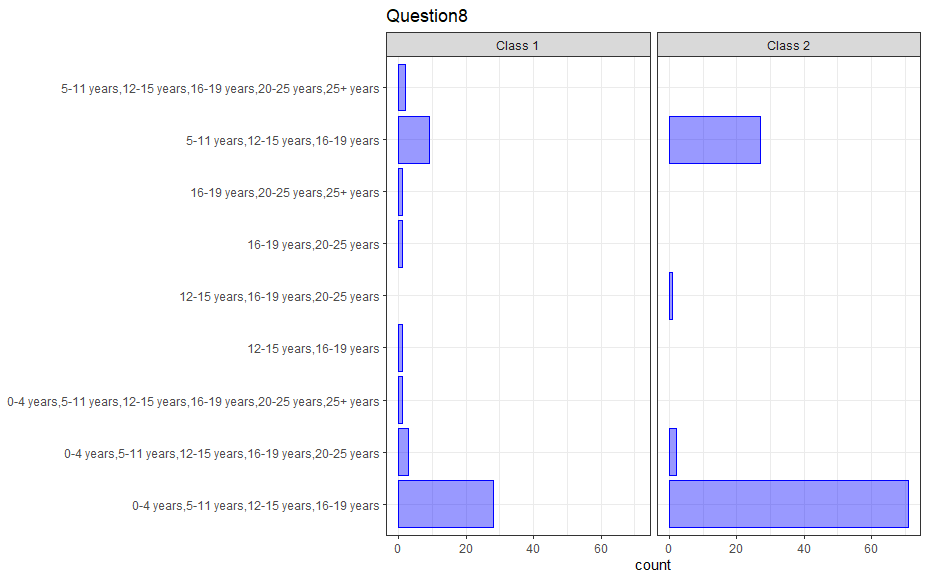


### Question 9: At what age do children and young people with behaviours that challenge transition out of the service to a service for individuals who are older?

This question shows the range of ages that children and young people with challenging behaviour transition out of the service. Figure S7 shows the distribution of responses from the original survey.

It is apparent from the data that the vast majority of services have individuals transition from the service at age 18. The variable was collapsed into fewer categories to attempt to keep this variable within the model but this did not add anything to the model, so was removed.

Figure S7 presents the patterns of responses for Question 9.


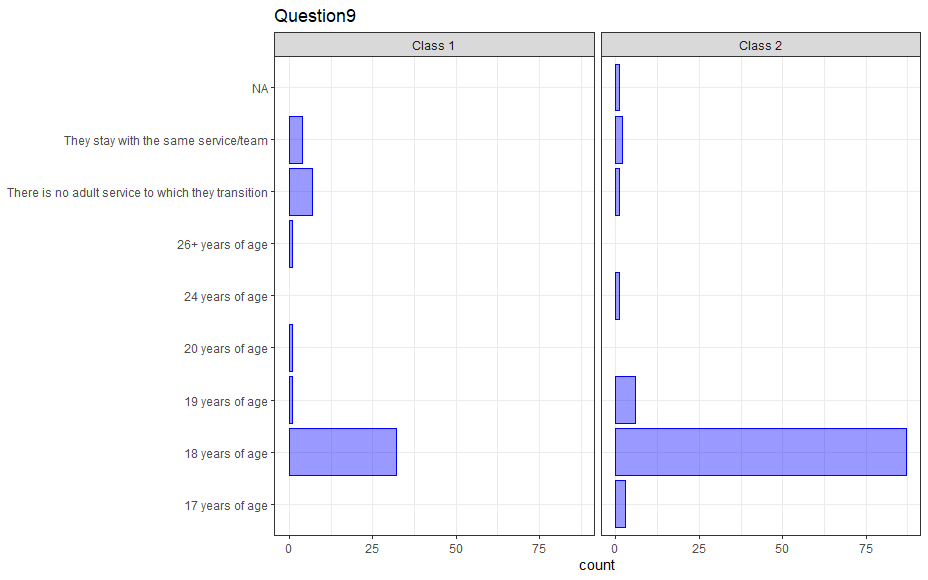


### Question 10: For those children and young people with behaviours that challenge transitioning out of the service, to what service(s) do they transition?

This question allowed the services to check four options: community adult learning disability service; community forensic services; Adult intensive support team/ behaviours support team; or Other. In general the pattern of responses was relatively uninformative as most included “Community adult learning disability services” with some combination of other teams in smaller proportions (Figure S8).

Figure S8 shows the distribution of responses for question 10.


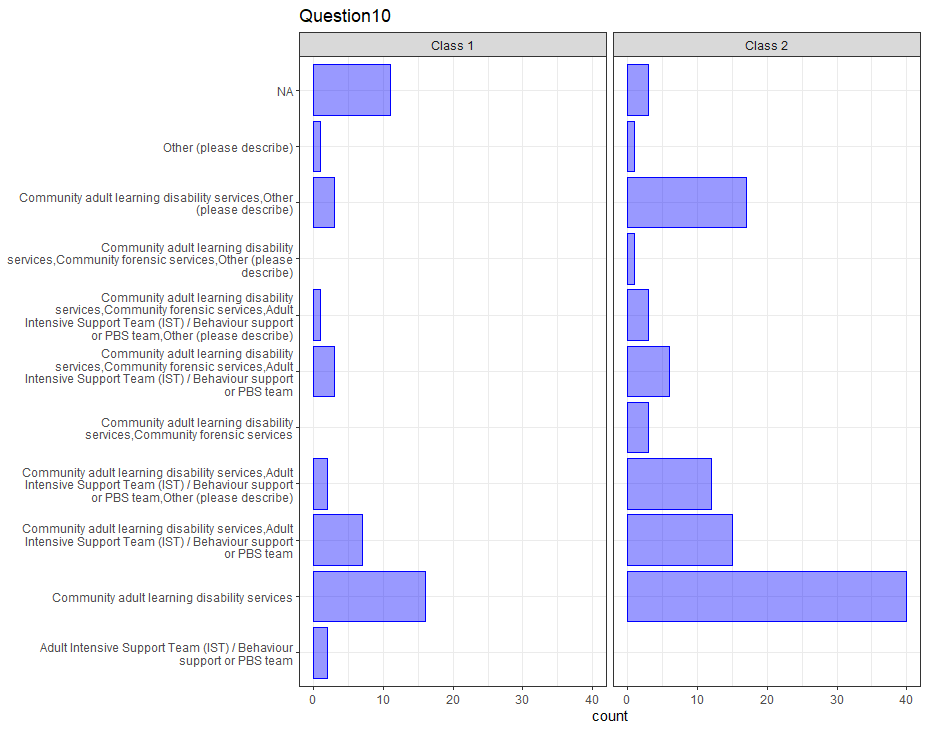


### Question 15: In a typical year, how many referrals to the service are received (new and re-referrals)?

This question posed a problem for the statistical analysis as the variable was highly skewed and contained outliers. This skew would bias the analysis considerably. A provisional run of the model did include this variable, but it showed little additional distinction in the model (Figure S9).

Figure S9 shows the distribution of responses for question 15.


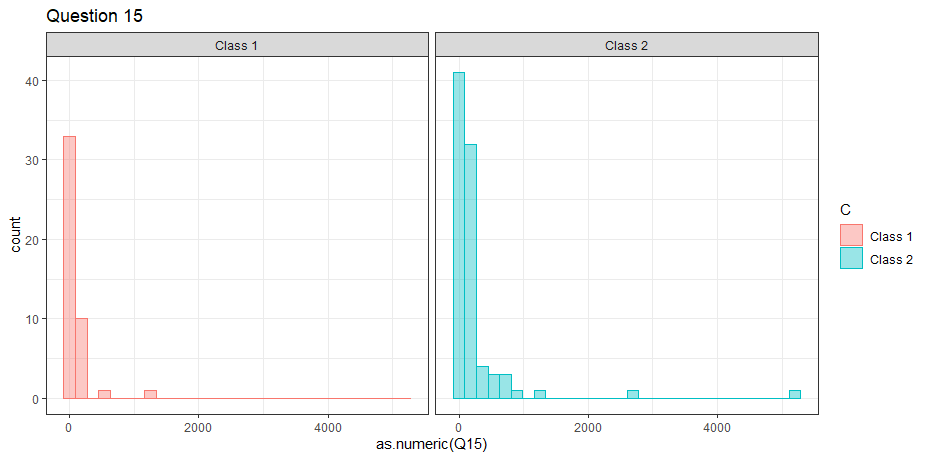


### Question 24: What intervention approaches does your service typically carry out that involve directly delivering interventions to children and young people with behaviours that challenge?

The types of interventions that a service offered were recorded in question 24. There were over 90 unique patterns of responses that were categorized by the qualitative team into three plausible response categories that might show some distinction. It was found that this grouping showed only a few responses that were not offering most intervention categories, so this item showed little distinction between the services and was removed (Figure S10).

Figure S10 shows the pattern of responses for Question 24.


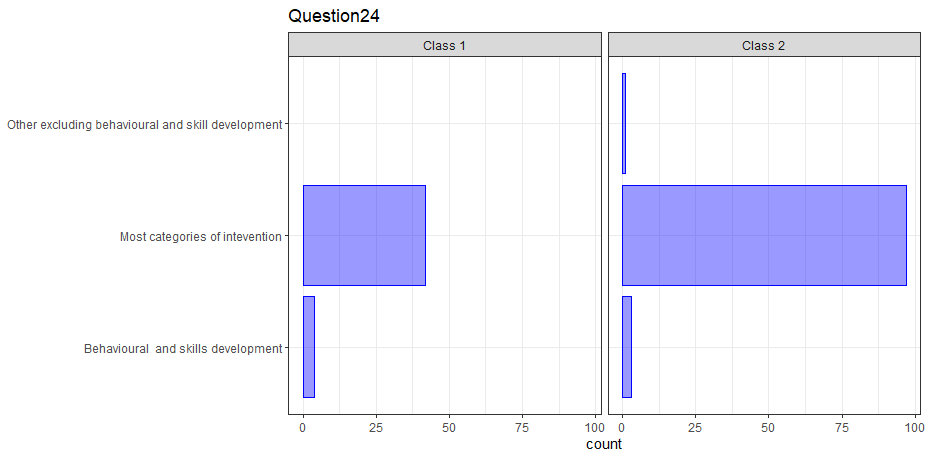


### Question 25: What supports for children and young people with behaviours that challenge does your service typically use that involve supporting others to deliver interventions

This question has been classed as having too sparse information indicating that the number of unique patterns of response was very large, showing very little similarities and making grouping very complex to achieve without a massive amount of data to base the groupings. Summary of this information was not practically possible as most frequencies of response were N=1, and plots were too big or not possible.

### Question 32: What methods does the service regularly use to obtain feedback about the service from children and young people with behaviours that challenge and their families and others?

This question was not considered to be useful in providing discrimination between services, so was omitted prior to the analysis. I t was retained in the survey as a useful descriptive variable for the qualitative evaluation. The data was not recategorized, so similarly to the Question 25, had more than seventy unique response patterns which made creating summaries complex and relatively uninformative, so have not been included for brevity.

**Section 3 Descriptive analysis**

***Descriptive analysis process***

1. The research data and services database were exploratively reviewed for information
2. The initial framework was then shared with the Chief Investigator for feedback and refinement. The feedback at this stage focused on describing and refining distinctions of each potential service model.
3. A full review of survey data and interview data was then carried out to refine the initial framework of service models. Online searches were also conducted to find additional information (where possible) about services to support this process.
4. The research team met again to discuss and finalised a framework of service models.
5. The framework was shared again with the Chief Investigator for final feedback and refinement. The Chief Investigator and a member of the research team discussed potential service models and distinctions within each service model. These discussions also focused on services which did not appear to naturally fit into a particular model.
6. One final review of collected data and online searches was then undertaken to review small groups of or individual services where queries were raised about where they were best classified.
7. The finalised framework of service models was then used by one member of the research team to classify services, with another member of the research team reviewing all classifications to ensure agreement. Survey data were used primarily to classify services, with interview data only used when further distinction was required. For example, it was unclear from one service’s survey data how they related to the wider intellectual disability service and their interview data clarified this relationship, allowing the research team to appropriately classify the service. From survey data for another service, it was not clear whether they were an ID-CAMHS or a generic CAMHS and interview data confirmed that they were an ID-CAMHS.
8. Consultation was sought from the MELD Study Family Carer Advisory Group, Professional Advisory Group, Study Management Group, and Study Steering Committee; all of whom confirmed the face validity of the groupings of service models within the framework.
9. Further reviews were undertaken by the research team to review all classifications of services. This included finding additional information online and checking with services themselves where necessary.

# Section 4 Information on Items for LCA analysis

Table S2: Items for LCA analysis

| **Variable** | **Variable type**  **(No. of categories)** | **Description**  **(Original survey question)** | **Variable manipulation from original survey questions (recategorized or transformed)** | **Probability of response in each class from 2-class LCA (CATEGORICAL) OR Mean/SE for continuous items.** |
| --- | --- | --- | --- | --- |
| **Q5** | Multiple dichotomous (5) | Q5 Who commissions the service? If this service is jointly commissioned, please select all that apply. If there is more than one commissioner, you will then be asked to explain the rough proportions of share in funding.   - NHS specialist commissioning - Local authority: Education - Local authority: Social care - Private organization or company - Voluntary organization or charity - Private individual - Clinical commissioning group (CCG) - Transforming care partnership (TCP) - Integrated care system (ICS) - Sustainable transformation partnership (STP) - Other (text from ‘other’ answer in Q5): | Convert to multiple items:   1. single vs multiple commissioner 2. dichotomous items -   These items capture the types of commissioner:   1. Any Local authority (including social care or education or both) (yes/no) 2. Any Clinical Commissioning Group (CCG) (yes/no) 3. Any Transforming Care Partnership (TCP)/Integrated Care System (ICS)/ Provider collaborative(yes/no) 4. Any NHS England specialist commissioning (yes/no) | Class 1 (32.47%) Class 2 (67.53%)  Q5B1_1 0.558 0.940  Q5B1_2 0.442 0.060  Q5B2_1 0.428 0.026  Q5B2_2 0.572 0.974  Q5B3_1 0.856 0.938  Q5B3_2 0.144 0.062  Q5A, and Q5B4 – omitted from the analysis. |
| **Q11** | Categorical (2) | What groups of children and young people with behaviours that challenge is the service for (in terms of inclusion criteria for the service)? Please select all that apply   - Children and young people with learning (intellectual) disabilities - Children with global developmental delay - Autistic children and young people who do not have a learning (intellectual disability) - Children and young people who both have learning (intellectual) disabilities and who are also autistic - Other disabled children and young people - Non-disabled children and young people - Other children and young people with particular “diagnoses” (please describe if selected): | This variable was reclassified into the following three categories:   1. Learning (intellectual) disabilities AND/OR Autism only 2. Learning (intellectual) disabilities and Autism and Other | Class 1 (32.47%) Class 2 (67.53%)  Q11_1 0.617 0.516  Q11_2 0.383 0.484 |
| **Q12** | Dichotomous | Is the service only for children and young people with behaviours that challenge (even if they also have other support needs)?  Yes \| No | Unchanged from raw survey data   1. No 2. Yes | Class 1 (32.47%) Class 2 (67.53%)  Q12_1 0.330 0.869  Q12_2 0.670 0.131 |
| **Q14** | Dichotomous | Who can refer children and young people with behaviours that challenge into your service? Please select all that apply   - General Practitioners/Primary Care - Self-referrals/Referral directly from the child’s carer - School – mainstream - School - special - Child and Adolescent Mental Health Services (CAMHS) - Other health professionals - Pre-schools - Social services - Third sector organisations - Paediatricians - Other (please describe) | Information from this question has been re-categorised by the research team into a dichotomous variable:  “Does the service accept self-referrals as well as professional referrals?”   1. No, Professional referral only 2. Yes, Professional or Self-referral | Class 1 (32.47%) Class 2 (67.53%)  Q14_1 0.797 0.282  Q14_2 0.203 0.718 |
| **Q21/22** | Categorical (x2) | Q21 Do any of the staff in the service have any specialist training and qualifications (with some certification such as University or other training provider awards/credits) in behaviours that challenge beyond their professional training? For example, in positive behaviour support?  Yes \| No | These variables will be reconfigured into three categorical variables indicating their intensity of expertise on each of the three domains from Q22:   - Positive behaviour support course   Categories for each variable will be as follows:   1. No staff with specialist training or less than 50% have specialist training 2. 50-75% of staff have specialist training 3. >75% have specialist training | Class 1 (32.47%) Class 2 (67.53%)  Q22A_1 0.481 0.837  Q22A_2 0.166 0.071  Q22A_3 0.354 0.092 |
|  |  | Q22 How many staff in the service have had specialist training and qualifications in behaviours that challenge beyond their professional training? Please enter total number of staff for all that apply. If no staff have these qualifications please enter 0 (zero). *(only displayed if ‘yes’ selected in Q21)*   - Positive behaviour support course - Challenging behaviour course - Other (please describe) | These variables will be reconfigured into three categorical variables indicating their intensity of expertise on each of the three domains from Q22:   - Challenging behaviour course   Categories for each variable will be as follows:   1. No staff with specialist training or less than 50% have specialist training 2. >50% of staff have specialist training | Class 1 (32.47%) Class 2 (67.53%)  Q22B_1 0.731 0.968  Q22B_2 0.269 0.032 |
| **Q31** | Dichotomous | What outcome domains does your service typically measure for children and young people with behaviours that challenge and their families? Please select all that apply  The child and/or young person's behaviours that challenge  The child and/or young person's quality of life  The child and/or young person’s skills, such as communication skills, social skills, independence skills  The child and/or young person's mental health (e.g., anxiety, mood/depression)  Family carer well-being and quality of life (including quality of life of the family as a whole)  Family carer experience and satisfaction with services/support received  Other (please describe) | Information from this question has been re-categorised by the research team into a dichotomous variable:   1. Child measures only 2. Child and parent\|family measures | Class 1 (32.47%) Class 2 (67.53%)  Q31_1 0.979 0.879  Q31_2 0.021 0.121 |

**Section 5 - MELD Study survey**

We would like to collect the following information about your service for the study: Mapping and Evaluating Services for Children with Learning Disabilities and Behaviours that Challenge (MELD). **Please note:** where exact figures are unable to be given, please provide us with approximate values.

Name of Service: ________________________________________________

**Q1 How long has the service been in place?**

**Years Months**

**Q2 Is the service a temporary or short term service/project with a fixed end date?**
☐ Yes ☐ No

If yes, please describe:

**Q3 Does the service stand-alone or is it part of/a sub-team of another service?**

☐ Stand-alone service ☐ Part of another service  *If you are a stand-alone service, please skip to question 5.*

**Q4 Please describe what other service or sub-team your service is part of:**

**Q5 Who commissions the service? If this service is jointly commissioned, please tick all that apply.**

| ☐ NHS England specialist commissioning | ☐ Private individual |
| --- | --- |
| ☐ Local Authority: Education | ☐ Clinical Commissioning Group (CCG) |
| ☐ Local Authority: Social Care | ☐ Transforming Care Partnership (TCP) |
| ☐ Private organisation or company | ☐ Integrated Care System (ICS) |
| ☐ Voluntary organisation or charity | ☐ Sustainable Transformation Partnership (STP) |
| ☐ Other (please describe): |  |

|  |
| --- |
|  |
|  |
|  |
|  |
|  |
|  |
|  |
|  |
|  |

**Q6 *If you selected only one commissioner, please skip to question 7.* Otherwise, please tell us the approximate percentage of funding that comes from each of the options you selected**
NHS specialist commissioning :

Local authority: Education :

Local authority: Social care :

Private organisation or company :

Voluntary organisation or charity :

Private individual :

Clinical commissioning group (CCG) :

Transforming care partnership (TCP) :

Integrated care system (ICS) :

Sustainable transformation partnership (STP) :

Other (*if you answered ‘other’ to Q5*):

**Q7 What are the ages of the children and young people with behaviours that challenge who can access the service? Please tick all that apply**

☐ 0-4 years ☐5-11 years ☐ 12-15 years ☐16-19 years ☐ 20-25 years ☐25+ years

**Q8 At what age do children and young people with behaviours that challenge transition out of the service to a service for individuals who are older?**

years

☐ There is no adult service to which they transition to
☐ They stay with the same service/team

*If you answered ‘there is no adult service to which they transition to’ or ‘they stay with the same service/team’ please skip to question 10.*

**Q9 For those children and young people with behaviours that challenge transitioning out of the service, to what service(s) do they transition? Please tick all that apply**.

☐Community adult learning disability services
☐ Community forensic services

☐ Adult Intensive Support Team (IST)/ Behaviour support or PBS team

☐ Other (please describe)

**Q10 What groups of children and young people with behaviours that challenge is the service for (in terms of inclusion criteria for the service)? Please tick all that apply**
☐ Children and young people with learning (intellectual) disabilities

☐ Children with global developmental delay

☐ Autistic children and young people who do not have a learning (intellectual disability)

☐ Children and young people who both have learning (intellectual) disabilities and who are
 also autistic

☐ Other disabled children and young people
☐ Non-disabled children and young people
☐ Other children and young people with particular “diagnoses” (please describe):

**Q11 Is the service only for children and young people with behaviours that challenge (even if they also have other support needs)?**

☐Yes ☐No *If you selected yes, please skip to question 13.*

**Q12 If you said that the service is not exclusively for children and young people with behaviours that challenge. Please describe who else/what other groups the service is for.**

**Q13 Who can refer children and young people with behaviours that challenge into your service? Please tick all that apply**

☐General Practitioners/Primary Care ☐Pre-schools
☐Self-referrals/Referral directly from the child’s care ☐Social services
☐School – mainstream ☐Third sector organisations

☐School- special ☐Paediatricians
☐Child and Adolescent Mental Health Services (CAMHS) ☐Other health professionals

☐Other (please describe):

**Q14 In a typical year, approximately how many referrals to the service are received (new and re-referrals)?**

**Q15 Approximately what is the current total active caseload for the service of children and young people with behaviours that challenge?**

**Q16 For each of the following professional groups, please indicate how many staff in the service are from this background and the approximate full time equivalent for each group. If nobody of this profession works in the service, please answer ‘0’. Please categorise staff by main role. If there are staff in your service from other backgrounds, please indicate these in the ‘other care staff’ option.**

| **Professional Group** | **Number of staff** | **Full time equivalent** |
| --- | --- | --- |
| Assistant Psychologist |  |  |
| Assistant Social Worker |  |  |
| Clinical Psychologist |  |  |
| Dietician |  |  |
| General Nurse |  |  |
| Health Care Assistant |  |  |
| Learning Disability Nurse |  |  |
| Mental Health Nurse |  |  |
| Occupational Therapist |  |  |
| Physiotherapist |  |  |
| Psychiatrist (e.g., Consultant Psychiatrist, Staff Grade Doctor) |  |  |
| Qualified teacher |  |  |
| Speech and Language Therapist |  |  |
| Social Worker |  |  |
| Support worker |  |  |
| Other (please describe): |  |  |

**Q17 Do any of the staff in the service have any specialist training and qualifications (with some certifications such as University or other training provider awards/credits) in behaviours that challenge beyond their professional training? For example, in positive behaviour support?**

☐Yes ☐No

*If you selected no, please skip to question 19.*

**Q18 How many staff in the service have had specialist training and qualifications in behaviours that challenge beyond their professional training? Please enter total number of staff for all that apply.**

|  |
| --- |
|  |

Positive behaviour support course

Challenging behaviour course

**Q19 What assessment approaches does your service typically carry out for referrals of children and young people with behaviours that challenge? *Please tick all that apply***

| ☐General health screen/assessment | ☐Functional assessment of challenging behaviour |
| --- | --- |
| ☐Medicines/drugs review | ☐Sensory assessment |
| ☐Mental health assessment | ☐Cognitive assessment |
| ☐Communication assessment | ☐Autism assessment |
| ☐Other (please describe): |  |

**Q20 What intervention approaches does your service typically carry out that involve directly delivering interventions to children and young people with behaviours that challenge? *Please tick all that apply***

☐ Reducing challenging behaviour using medication

☐ Behavioural interventions that concern behaviours that challenge

☐ Writing a behaviour support plan

☐ Delivering a multi-element behaviour support

☐ Increasing communication skills
☐ Increasing other adaptive skills (e.g., social or independence skills)

☐ Psychological therapies for mental health problems

☐ Physical health interventions

☐ Pharmacological interventions for mental health problems

☐ Psychological / pharmacological interventions for poor sleep

☐ Other therapies (e.g., art therapy, music therapy, play therapy)

☐ Sensory interventions

☐ Other (please describe):

**Q21 What supports for children and young people with behaviours that challenge does your service typically use that involve supporting others to deliver interventions (e.g., training)? *Please tick all that apply***

☐Mentoring/supervising other staff to deliver a behaviour support plan

☐Training in assessment and intervention for challenging behaviour

☐Training family carers

☐Training paid carers

☐Consulting on individual cases

☐Providing resources for families to use at home

☐Parenting programmes

☐Wellbeing interventions for families

☐Other (please describe):

**Q22 What outcome domains does your service typically measure for children and young people with behaviours that challenge and their families? *Please tick all that apply***

☐The child and/or young person's behaviours that challenge

☐The child and/or young person's quality of life

☐The child and/or young person's skills, such as communication skills, social skills,
 independence skills

☐The child and/or young person's mental health (e.g., anxiety, mood/depression)

☐Family carer well-being and quality of life (including quality of life of the family as a whole)

☐Family carer experience and satisfaction with services/support received

☐Other (please describe):

**Q23 What methods does the service regularly use to obtain feedback about the service from children and young people with behaviours that challenge and their families and others? *Please tick all that apply***

☐Interviews with children and young people

☐Interviews with family carers

☐Interviews with other staff

☐Focus groups with children and young people

☐Focus groups with family carers
☐Focus groups with other staff
☐Advisory groups with children and young people
☐Advisory groups with family carers
☐Advisory groups with other staff
☐Service satisfaction questionnaires with children and young people
☐Service satisfaction questionnaires with family carers
☐Service satisfaction questionnaires with other staff
☐Professionals and staff from other agencies
☐None
☐Other (please describe):

**Section 6 - MELD Study interview schedule**

**History of the service, and upcoming changes**

In the online survey, we asked questions about how long the service has been in place, but I wondered if you could tell me what services existed in the local area before the service started up?

Could you please tell me why the service was established?

Are there any changes being planned or implemented in the service in response to new funding?

*Prompts: Admission Avoidance, Other Innovation Calls*

**Personnel**

Please could you describe the management and supervision structure within the service?

*Prompts: Who are the heads of the service? Is there a clinical lead and a management lead separately? Do people take a lead in particular areas of practice? What supervision arrangements are in place? Is supervision separated by professional group?*

Are there any sub-teams within your service?

*Prompts: North/South sub-teams within the region the service covers?*

If yes, please can you describe what the sub-teams within your service are?

**Interconnections**

Does your service regularly work in partnership with any other services to deliver supports to children and young people with learning disability and behaviours that challenge and their families?

If yes, please can you describe who the partners are and the nature of the partnership?

*Prompts: Other NHS Services, Local Authority, Education, Special Schools, Charity, Private Sector*

**Accessing the service**

In the online survey, we asked questions about the access criteria about age and diagnoses. The responses were [Researcher summarises the responses to these questions]

Are there any other access/eligibility criteria for the service?

In the online survey, we asked specifically about translation services offered for people who do not speak English, or speak English as an additional language. We are also interested in any other ways in which the service addresses cultural differences in the way children and young people with behaviours that challenge and families are supported.

Does your service have any particular methods of ensuring sensitivity to the religion and/or culture of the children and young people with behaviours that challenge and their families you work with?

If yes, please can you describe these?

**Involvement and co-production:**

In the online survey, we asked questions about how your service gathers feedback. Following on from that, I wondered whether there were opportunities for children and young people with behaviours that challenge and their families to be engaged or involved in decisions made about the service, for example in service development, monitoring, or in an advisory capacity?

If yes, please can you describe this?

In this study, we are defining co-production as a way of working where service providers and the people who use them work together to produce shared outcomes

*Prompts: co-design of services and supports, co-delivery of services to other families, development of informative and user-friendly service information, interviews for new staff, developing pathways.*

I wondered whether your service undertakes co-production work with children and young people with learning disability and behaviours that challenge and/or their families?

If yes, please could you describe the co-production work?

# References

1. Weller BE, Bowen NK, Faubert SJ. Latent class analysis: A guide to best practice. Journal of Black Psychology. 2020;46(4):287–311.
